# A Genome-Wide Association Study Identifies Potential Susceptibility Loci for Hirschsprung Disease

**DOI:** 10.1371/journal.pone.0110292

**Published:** 2014-10-13

**Authors:** Jeong-Hyun Kim, Hyun Sub Cheong, Jae Hoon Sul, Jeong-Meen Seo, Dae-Yeon Kim, Jung-Tak Oh, Kwi-Won Park, Hyun-Young Kim, Soo-Min Jung, Kyuwhan Jung, Min Jeng Cho, Joon Seol Bae, Hyoung Doo Shin

**Affiliations:** 1 Research Institute for Basic Science, Sogang University, Seoul, Republic of Korea; 2 Department of Life Science, Sogang University, Seoul, Republic of Korea; 3 Department of Genetic Epidemiology, SNP Genetics, Inc., Seoul, Republic of Korea; 4 Department of Computer Science, University of California Los Angeles, Los Angeles, California, United States of America; 5 Division of Pediatric Surgery, Department of Surgery, Samsung Medical Center, Sungkyunkwan University School of Medicine, Seoul, Republic of Korea; 6 Department of Pediatric Surgery, Asan Medical Center, University of Ulsan College of Medicine, Seoul, Republic of Korea; 7 Department of Pediatric Surgery, Severance Children’s Hospital, Yonsei University College of Medicine, Seoul, Republic of Korea; 8 Department of Pediatric Surgery, Seoul National University Children’s Hospital, Seoul, Republic of Korea; 9 Department of Surgery, Seoul National University Bundang Hospital, Seongnam, Gyeonggi, Republic of Korea; 10 Department of Surgery, Konkuk University Medical Center, Seoul, Republic of Korea; 11 Laboratory of Translational Genomics, Samsung Genome Institute, Samsung Medical Center, Seoul, Republic of Korea; University of Hong Kong, Hong Kong

## Abstract

Hirschsprung disease (HSCR) is a congenital and heterogeneous disorder characterized by the absence of intramural nervous plexuses along variable lengths of the hindgut. Although *RET* is a well-established risk factor, a recent genome-wide association study (GWAS) of HSCR has identified *NRG1* as an additional susceptibility locus. To discover additional risk loci, we performed a GWAS of 123 sporadic HSCR patients and 432 unaffected controls using a large-scale platform with coverage of over 1 million polymorphic markers. The result was that our study replicated the findings of *RET*-*CSGALNACT2*-*RASGEF1A* genomic region (*_raw_P* = 5.69×10^−19^ before a Bonferroni correction; *_corr_P* = 4.31×10^−13^ after a Bonferroni correction) and *NRG1* as susceptibility loci. In addition, this study identified *SLC6A20* (*_adj_P* = 2.71×10^−6^), *RORA* (*_adj_P* = 1.26×10^−5^), and *ABCC9* (*_adj_P* = 1.86×10^−5^) as new potential susceptibility loci under adjusting the already known loci on the *RET*-*CSGALNACT2*-*RASGEF1A* and *NRG1* regions, although none of the SNPs in these genes passed the Bonferroni correction. In further subgroup analysis, the *RET*-*CSGALNACT2*-*RASGEF1A* genomic region was observed to have different significance levels among subgroups: short-segment (S-HSCR, *_corr_P* = 1.71×10^−5^), long-segment (L-HSCR, *_corr_P* = 6.66×10^−4^), and total colonic aganglionosis (TCA, *_corr_P*>0.05). This differential pattern in the significance level suggests that other genomic loci or mechanisms may affect the length of aganglionosis in HSCR subgroups during enteric nervous system (ENS) development. Although functional evaluations are needed, our findings might facilitate improved understanding of the mechanisms of HSCR pathogenesis.

## Introduction

Hirschsprung disease (HSCR, or aganglionic megacolon) is a rare congenital disease (1 in 5000 live births) that leads to intestinal obstruction or chronic constipation. HSCR manifests a sex-dependent penetrance (male:female ratio of ∼4∶1) and involves mostly sporadic cases; however, 5–20% are familial forms. Based on the length of aganglionosis, patients can be further classified into three groups as follows: (1) short-segment (S-HSCR, ∼80% of cases) with aganglionosis affecting the rectum and not extending beyond the upper sigmoid, (2) long-segment (L-HSCR, ∼15%) with aganglionosis affecting longer tracts of the colon, and (3) total colonic aganglionosis (TCA, ∼5%) [1], [2]. Genetic variations in eight genes, including *RET*, have been implicated in less than 30% of HSCR development, indicating the need to identify additional HSCR-causing variations.

The *RET* proto-oncogene, encoding a tyrosine-kinase receptor, has been identified as the major HSCR gene. In particular, a common variant rs2435357 (also known as RET+3) within a conserved enhancer-like sequence in intron 1 of *RET* showed a significant association with HSCR susceptibility, with different genetic effects between male and female patients [3]. Since this variant is located in an enhancer-like sequence, it was further speculated that additional factors might interact with this variant to affect *RET* expression. Two downstream genes that are near *RET*, *CSGALNACT2* and *RASGEF1A*, are differentially expressed between the colon and small intestine [3], although the functions of these genes have not been adequately characterized.

Mutations in other genes (*EDNRB*, *GDNF*, *NRTN*, *SOX10*, etc.) have also been identified as contributing to HSCR development [1], [2], [4], [5]. Recently, the first genome-wide association study (GWAS) of HSCR has additionally identified *neuregulin 1* (*NRG1*), one of the molecular regulators in the development and maintenance of the enteric nervous system (ENS), as a susceptibility locus for HSCR [6]. Follow-up studies have also evaluated the potential effects of NRG1 on HSCR, including aberrant *NRG1* expression [7], [8]; however, results from these studies suggest that other alleles or epigenetic factors might affect *NRG1* expression. Moreover, still other factors (for instance, *APOB*, *RELN*, *GAL*, etc.) have been proposed that may be associated with HSCR development [9], [10].

Given that HSCR is a heterogeneous disorder, new confounders with small-to-modest effects on HSCR development have yet to be discovered. *RET* coding sequence mutations account for about 50% in familial HSCR and 15–20% in sporadic HSCR [1]. In this study, we have performed GWAS using sporadic HSCR to discover additional risk loci and to validate previous discoveries using a larger number of single nucleotide polymorphism (SNP) markers.

## Subjects and Methods

### Study subjects

Study subjects were collected from Samsung Medical Center, Asan Medical Center, Severance Children’s Hospital, and Seoul National University Children’s Hospital in Korea. The study protocol was approved by the Institutional Review Board of each hospital (IRB No. SMC_2010-02-028-003 of Samsung Medical Center; 2010-0395 of Asan Medical Center; 4-2010-0436 of Severance Children’s Hospital; 1006-129-322 of Seoul National University Children’s Hospital), and guardians of all subjects provided written informed consent. All subjects were of Korean ethnicity. A total of 124 sporadic HSCR patients (102 males and 22 females) were recruited, and their biopsy specimens or surgical materials were used to make the diagnosis of HSCR by histological examination based on the absence of the enteric ganglia. Patients were composed of 76 S-HSCR, 31 L-HSCR, and 17 TCA. For the controls, 450 unaffected subjects (250 males and 200 females) with no history of HSCR based on the questionnaire information about concomitant disease, but without exclusion criteria for other neurological diseases or gastroenterological diseases, were included from Ansan cohort provided by Korea BioBank, Center for Genome Science, National Institute of Health, Korea Centers for Disease Control and Prevention. However, 19 subjects (1 S-HSCR patient and 18 controls) were excluded from all analyses because they were detected as outliers based on the principal component analysis (PCA) and revealed as mixed-bloods.

### Genome-wide genotyping

Genomic DNAs were extracted from the peripheral blood lymphocytes of the patients and unaffected controls, using the Wizard Genomic DNA Purification Kit (Promega, WI, USA), according to the manufacturer’s protocol. A whole-genome genotype scan was performed using about 200 ng of the genomic DNAs on Illumina’s HumanOmni1-Quad BeadChip (Illumina, San Diego, USA), according to the manufacturer’s protocol. All samples were scanned using the Illumina iScan system (Illumina), and the normalized bead intensity data were loaded into the GenomeStudio software (Illumina). Considering a potential association between rare variants and rare diseases such as HSCR, this study included all polymorphic markers for association analysis. For 1,140,419 markers on the chip, SNP marker quality control (QC) was applied as follows: (1) only SNP with call rate (>98%, 995,666 markers) in both cases and controls was included, (2) 221,556 monomorphic markers were excluded, and (3) visual inspection of the genotype cluster image was performed for the SNPs with deviation from Hardy-Weinberg equilibrium (HWE, *P*<0.0001), and 16,850 SNPs deviating from HWE were excluded from the analysis. The 757,260 markers that remained after QC were ultimately used for further association analysis.

### Statistics

Associations of genotype distributions were calculated by logistic regression analysis using HelixTree software (Golden Helix, Bozeman, MT, USA). The possible population stratification was examined by PCA using HelixTree software. A Bonferroni correction applied by the number of independent SNPs (757,260), which resulted in the threshold of GWAS significance (*_corr_P* = 6.6×10^−8^), was calculated. In order to remove the effects of primary top signals from the known *RET*-*CSGALNACT2*-*RASGEF1A* genomic region on chromosome 10q11.2 and the effect of the first GWAS-discovered *NRG1* on chromosome 8p12, four SNPs, including rs2435357, rs1800860, and rs7078220 on *RET*-*CSGALNACT2*-*RASGEF1A* region and rs16879552 on *NRG1*, were adjusted as covariates. In the case of the three SNPs on *RET*-*CSGALNACT2*-*RASGEF1A* region, they were likely to make a major contribution to the strongest signal according to our GWAS results. Therefore, *P*-values were defined as follows: *_raw_P* for raw *P*-value before a Bonferroni correction, *_corr_P* after a Bonferroni correction, and *_adj_P* under adjusting the known *RET*-*CSGALNACT2*-*RASGEF1A* and *NRG1* loci. The FaST-LMM (Factored Spectrally Transformed Linear Mixed Models) program was applied to correct for hidden relatedness [11]. Statistical power of the sample size was calculated using the Power for Genetic Association Analyses (PGA) software [12], with false positive rate of 5%, disease prevalence of 0.02% (1 in 5000 live births) [1], given minor allele frequencies of the most significant allele and sample sizes of case (n = 123) and control (n = 432), and assuming a relative risk of 1.5, resulting in the statistical power = 58.5%. Analysis of linkage disequilibrium (LD) among the SNPs was performed using the Haploview v4.2 software downloaded from the Broad Institute (http://www.broadinstitute.org/mpg/haploview) based on LD coefficients (|*D'*| and *r^2^*) between all pairs of biallelic loci.

## Results

### Characteristics of study subjects

After excluding outliers based on the PCA (Figure S1), a total of 555 subjects (123 sporadic HSCR patients and 432 unaffected controls) were involved in the GWAS. Although our study subjects showed genomic control inflation factor (λ) of 1.077, this inflation factor was reduced to 1.015 after correcting for hidden relatedness using FaST-LMM. Male:female ratio was ∼4.9∶1, as was expected based on general sex differences in HSCR. Patients were classified into groups of 75 S-HSCR (60.1% of cases; 65 male, 10 female), 31 L-HSCR (25.2% of cases; 24 male, 7 female), and 17 TCA (13.8% of cases; 13 male, 4 female), indicating that the proportion of patients with L-HSCR and TCA in this study was slightly higher than the expected prevalence of each subgroup.

### Association analysis and identification of new susceptibility locus

**Figure 1:**
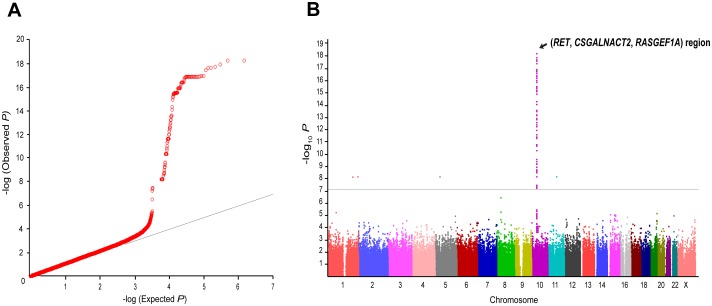
Overview of genome-wide association results. (A) Q-Q plot. The observed *P*-value (y-axis) is compared with the expected *_raw_P*-value (x-axis, under null distribution) before a Bonferroni correction. (B) Graphical summary (Manhattan plot) presenting *_raw_P*-values for the association with HSCR in 123 sporadic HSCR patients and 432 unaffected controls. The −log_10_ *P* (logistic regression analysis) is plotted against its physical position on successive chromosomes. Gray line represents the threshold for GWAS significance after a Bonferroni correction.

A quantile-quantile (Q-Q) plot for the association test with HSCR showed a significant deviation of measures at the tail (Figure 1A), even after excluding SNPs in the *RET*-*CSGALNACT2*-*RASGEF1A* region on chromosome 10q11.2 (Figure S2), indicating potentially true associations between the SNPs and HSCR. Our GWAS confirmed two facts previously reported. First, the strongest significant association was observed at the *RET*-*CSGALNACT2*-*RASGEF1A* genomic region (Figure 1B), with top signal at kgp4676284 (rs1864400, *_raw_P* = 5.69×10^−19^ before a Bonferroni correction; *_corr_P* = 4.31×10^−13^ after a Bonferroni correction, Table S1). Second, *NRG1* also showed association signals (Table S2).

**Figure 2:**
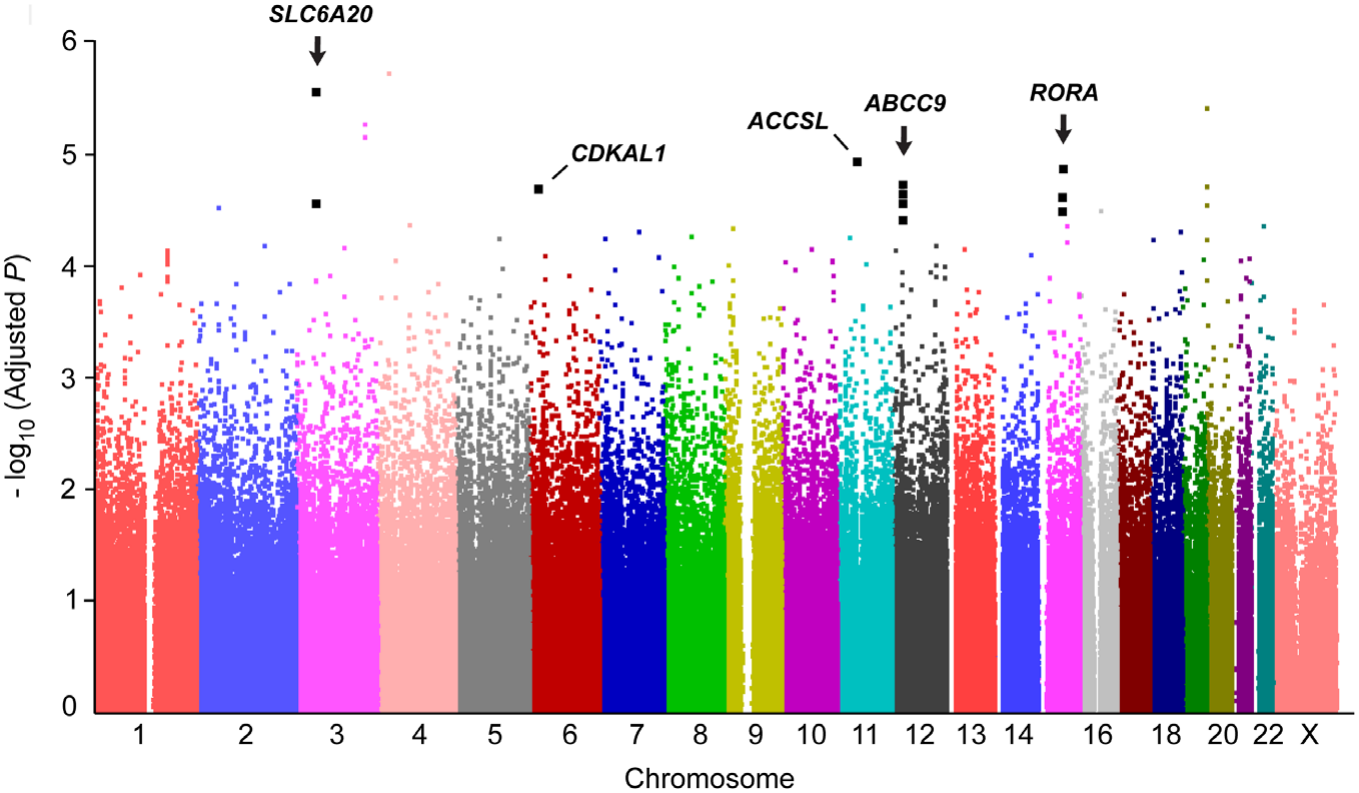
Result of the analysis adjusted by *RET*-*CSGALNACT2*-*RASGEF1A* and *NRG1* and top SNPs within the gene region. The *_adj_P*-value (y-axis) after adjustment by rs2435357, rs1800860, and rs7078220 on *RET*-*CSGALNACT2*-*RASGEF1A* region and rs16879552 on *NRG1* is plotted against its physical position on successive chromosomes.

**Figure 3:**
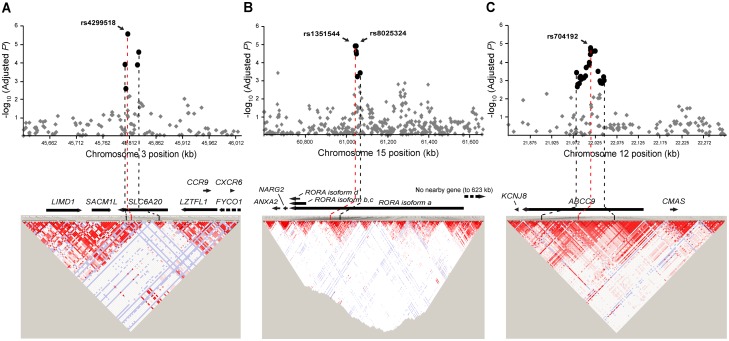
Regional association and LD plots of *SLC6A20*, *RORA*, and *ABCC9* SNPs for HSCR. Associations of SNPs across approximately (A) a 400 kb region around *SLC6A20* on chromosome 3p21.3, (B) a 1,065 kb region around *RORA* on chromosome 15q22.2, and (C) a 500 kb region around *ABCC9* on chromosome 12p12.1, under analysis adjusted by *RET*-*CSGALNACT2*-*RASGEF1A* and *NRG1*, are shown. In the upper panel of each regional association, strong associations are shown as large black circles; relatively weak associations are shown as small gray diamonds. In the lower panel, LDs are indicated with LD coefficient (*r^2^*) between all pairs of biallelic loci.

No other individual SNPs reached a genome-wide significance (Bonferroni-corrected significance) level, except for five SNPs (two intronic rs12739262 and rs35198051; three intergenic rs12752277, rs2809867, and rs36019094; Figure 1B and Table S3). To identify additional signals under exclusion of effects from the *RET*-*CSGALNACT2*-*RASGEF1A* region and from the first GWAS-discovered *NRG1*, further analysis was employed by adjusting for four SNPs, rs2435357, rs1800860, and rs7078220 on the *RET*-*CSGALNACT2*-*RASGEF1A* region (these three SNPs were confirmed to sufficiently represent the region) and rs16879552 on *NRG1*. The strongest association was detected at *SLC6A20* encoding solute carrier family 6, proline IMINO transporter, member 20 (rs4299518, *_adj_P* = 2.71×10^−6^; rs2159272, *_adj_P* = 2.66×10^−5^; Table 1 and Figure 2). In the regional association of 400 kb around *SLC6A20* on chromosome 3p21.3 (Figure 3A), seven SNPs of *SLC6A20* showed relatively robust association signals (minimum *_adj_P* = 2.71×10^−6^, Table S4). LD analysis revealed that *SLC6A20* SNPs showing potential associations with HSCR were likely to not be in LD with nearby genes (Figure 3A). In addition, *RORA* (minimum *_adj_P* = 1.26×10^−5^) and *ABCC9* (minimum *_adj_P* = 1.86×10^−5^), along with relatively strong regional associations (Figure 3B and 3C), were detected as potential risk loci for HSCR (Table 1 and Table S4).

**Table 1:** Top 20 SNPs under analysis adjusted by SNPs of *RET* region on chr. 10 and *NRG1* on chr. 8.

SNP	Chr.	Position	Minorallele	Location	(Closest) gene	MAF		*_adj_P*-value*(Adjusted analysis)	*_corr_P*-value**
						Case(n = 123)	Control(n = 432)		
rs11725593	4	27066038	A	Intergenic	(*STIM2*)	0.317	0.204	1.80×10^−6^	NS
rs4299518	3	45809273	C	Intron	*SLC6A20*	0.004	0.053	2.71×10^−6^	NS
rs6074578	20	191797	T	Intergenic	(*DEFB129*)	0.549	0.389	3.71×10^−6^	NS
rs12639288	3	167661136	A	Intergenic	(*LOC100509398*)	0.085	0.036	5.11×10^−6^	NS
rs13069589	3	167680850	A	Intergenic	(*LOC100509398*)	0.081	0.035	6.66×10^−6^	NS
rs12284962	11	44077227	A	Intron	*ACCSL*	0.041	0.121	1.13×10^−5^	NS
rs1351544	15	61042867	T	Intron	*RORA*	0.354	0.213	1.26×10^−5^	NS
rs8025324	15	61043378	A	Intron	*RORA*	0.354	0.213	1.26×10^−5^	NS
rs704192	12	22015114	T	Intron	*ABCC9*	0.390	0.251	1.86×10^−5^	NS
rs6078500	20	182013	C	Intergenic	(*DEFB128*)	0.549	0.398	1.86×10^−5^	NS
rs9348455	6	21043852	T	Intron	*CDKAL1*	0.362	0.249	1.96×10^−5^	NS
rs704191	12	22015022	A	Intron	*ABCC9*	0.390	0.253	2.16×10^−5^	NS
rs9920560	15	61047930	A	Intron	*RORA*	0.358	0.219	2.37×10^−5^	NS
rs4148669	12	22024093	C	Intron	*ABCC9*	0.386	0.253	2.60×10^−5^	NS
rs2159272	3	45829995	T	Intron	*SLC6A20*	0.472	0.360	2.66×10^−5^	NS
rs6033398	20	183222	T	Intergenic	(*DEFB128*)	0.541	0.391	2.70×10^−5^	NS
rs12466120	2	53587240	A	Intergenic	(*GPR75-ABS3*)	0.524	0.396	2.82×10^−5^	NS
rs8050612	16	51025933	A	Intergenic	(*LOC100652974*)	0.159	0.081	3.03×10^−5^	NS
rs7183955	15	61049569	C	Intron	*RORA*	0.358	0.220	3.09×10^−5^	NS
rs704190	12	22014473	C	Intron	*ABCC9*	0.362	0.249	3.74×10^−5^	NS

*P-value after adjustment by sex and 4 SNPs (rs2435357, rs1800860, and rs7078220 on/nearby *RET* and rs16879552 on *NRG1*) as covariates.

**P-value after the Bonferroni correction.

Chr., chromosome; MAF, minor allele frequency; NS, not significant.

In the further analysis under adjusting the known *RET*-*CSGALNACT2*-*RASGEF1A* and *NRG1* loci, other genes including *CDKAL1* (*_adj_P* = 1.96×10^−5^), *ACCSL* (*_adj_P* = 1.13×10^−5^), and *ASTN1* (*_adj_P* = 7.63×10^−5^) also showed potential associations with HSCR (Table 1 and Table S5).

### Analysis of HSCR subgroups

**Figure 4:**
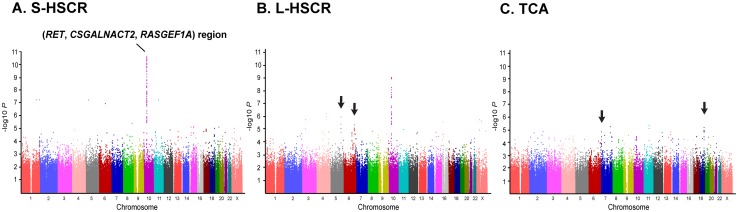
Manhattan plots of HSCR subgroup analysis. The strongest association *RET*-*CSGALNACT2*-*RASGEF1A* genomic region is observed at chromosome 10. Other potential loci are indicated by arrows. *P*-value indicates the significance before a Bonferroni correction. (A) short-segment (S-HSCR); (B) long-segment (L-HSCR); (C) total colonic aganglionosis (TCA).

In addition to *RET*, two of its downstream genes, *CSGALNACT2* and *RASGEF1A*, showed similar levels of significance regarding HSCR susceptibility and were in tight LD (Figure S3). In our further analysis among subgroups, this *RET*-*CSGALNACT2*-*RASGEF1A* genomic region showed different significance levels among subgroups (*_corr_P* = 1.71×10^−5^ in S-HSCR; *_corr_P* = 6.66×10^−4^ in L-HSCR; *_corr_P*>0.05 in TCA; Table S6 and Figure 4).

## Discussion

HSCR is a complex and heterogeneous disease. In addition, the incidence of HSCR is much higher in males, and a higher maternal inheritance than paternal (largely transmitted to the son) is observed in *RET* coding sequence mutations, based on the assumption of parent-of-origin effect [13]. Although mutations in *RET* (in particular, a common variant rs2435357 within a conserved enhancer-like sequence) and other genes (such as *GDNF*, *SOX10*, and endothelin-related genes) have partially accounted for HSCR development [3]–[5], [14], comprehensive genetic implications for HSCR and ENS development are still not fully understood. Inspired by the discovery of *NRG1* as an additional susceptibility locus from GWAS of HSCR, we performed another GWAS using a large-scale platform with coverage of over 1 million markers to identify additional risk factors and to investigate potential genetic effects among HSCR subgroups. As a result, this study identified *SLC6A20* and *ABCC9* as potential susceptibility loci and the possible reduced effects of the *RET*-*CSGALNACT2*-*RASGEF1A* genomic region according to length of aganglionosis.

Consistent with results of the first GWAS [6], we also replicated the finding that *NRG1* on chromosome 8 might be a susceptibility locus for HSCR (Figure 1B and Table S2). Due to the different GWAS array platforms, only *NRG1* rs16879552 in our GWAS was matched and could be compared to that of the previous GWAS. This study showed a nominal relevance at rs16879552 (Table S2), whereas the first GWAS revealed a higher association signal [6]. Since this study included no hereditary HSCR cases, this discrepancy between the Chinese and Korean studies might be due to ethnic differences among Asian populations or different inclusion ratios of the subgroups. However, among 41 *NRG1* SNP markers in our GWAS, several variants (for instance, rs7005606, rs4733130, and rs6996957) also showed increased association signals for HSCR (Table S2).

Five SNPs (rs12739262, rs12752277, rs2809867 on chromosome 1; rs36019094 on chromosome 5; rs35198051 on chromosome 11) showed genome-wide significance levels (Table S3). Among these SNPs, *PPP1R12B* rs12739262 and *SHANK2* rs35198051 (*_corr_P* = 0.005 for both SNPs) are positioned at the intron of the genes, whereas others are in intergenic or gene desert regions. However, despite potential associations of *SHANK2* with the nervous system and neurodevelopmental diseases [15], [16], no observation of additionally significant associations of *SHANK2* and *PPP1R12B* SNPs in our regional association results suggests that these loci with genome-wide significance might be false-positive findings. This study identified *SLC6A20*, *RORA*, and *ABCC9* as new potential susceptibility loci for HSCR. Four, nine, and nineteen SNPs of *SLC6A20*, *RORA*, and *ABCC9*, respectively, showed relatively robust association signals (*_adj_P*<0.001, Table S4), suggesting that these genes might play a role in HSCR susceptibility without the effects of the *RET*-*CSGALNACT2*-*RASGEF1A* and *NRG1* regions.

Although the functions of *SLC6A20* and its gene product are poorly understood, there are a few intriguing clues to the relationship between *SLC6A20* and HSCR. The human *SLC6A20* that is abundantly expressed in much of the gastrointestinal tract has been suggested as a target gene for a renal tubular disorder of iminoaciduria [17], [18]. Also, the combined mutations in *SLC6A20* and other genes have been observed to affect its related human phenotypes [19], suggesting that genetic variations of *SLC6A20* may also contribute to the development of the enteric system when combined with other risk factors. On the other hand, since *LIMD1*, a nearby gene of *SCL6A20*, has been shown to affect cell migration that is essential during development [20], we also analyzed LD between the seven *SLC6A20* SNPs (*_adj_P*<0.01) and two nearby genes (*LIMD1* and *SACM1L*) that were most likely to be in LD with *SLC6A20*. However, *SLC6A20* showed no LD with the nearby genes (Table S7), indicating that *SLC6A20* may have roles without correlation to nearby genes, but may interact with other regulators [21]. For other potential genes of *RORA* and *ABCC9*, no literature clues related to HSCR or ENS development could be found.

This study confirmed strong associations of SNPs on *CSGALNACT2* and *RASGEF1A* as nearby genes of *RET* and being in tight LD with one another. However, the functions of CSGALNACT2 and RASGEF1A are little understood. Only differential expressions of these three genes have been previously observed in human tissues: for instance, higher expression of *RASGEF1A* in the colon but lower in the small intestine, when compared to *RET* expressions [3]. Despite the insufficient sample size (in particular, the low number of L-HSCR and TCA cases), our results also identified other potential loci (Figure 4) that might contribute to the phenotypes, such as length of aganglionosis, of the L-HSCR and TCA subgroups. This evidence suggests that other regulators, including distant-acting factors or chromosomal abnormalities, may affect the development of ENS as well as HSCR [22], [23]. Therefore, further studies, including replications in large cohorts and deep sequencing of the *RET*-*CSGALNACT2*-*RASGEF1A* region in HSCR patients, are needed.

Notably, there are additional observations from this study. First, although the common *RET* rs2435357 within a conserved enhancer-like sequence has been evaluated to be associated with HSCR, possibly by affecting *RET* expression [3], [24], this study has found more significant associations at the top three intronic SNPs, kgp4676284 (rs1864400, *_corr_P* = 4.31×10^−13^, Table S1), kgp3302846 (rs741968, *_corr_P* = 4.31×10^−13^), and kgp11922846 (rs2742233, *_corr_P* = 9.54×10^−13^), which are in tight LD with each other but not in LD with rs2435357 (Figure S4). However, in our further in silico prediction analysis of potential branch point (BP) sites for alternative splicing using the Human Splicing Finder (http://www.umd.be/HSF/), these top three intronic SNPs did not emerge as potential BP sites. Second, in the case of *DSCAM* as a candidate gene in this study (Table S5), a most recent study has reported that this gene may be a predisposing locus in HSCR [25]. Therefore, further functional evaluations of these new observations may also be required.

To date, the association between *RET* genetic variations and HSCR has been identified in less than 30% of sporadic HSCR cases [1]. To investigate the genetic heterogeneity for genomic regions that showed the Bonferroni-corrected significances for HSCR, SNPs across approximately a 196 kb region around the *RET*-*CSGALNACT2*-*RASGEF1A* on chromosome 10 were further analyzed. As a result, this genomic region showed a relatively strong LD block (Figure S3). In addition, when a multidimensional scaling (MDS) of the *RET*-*CSGALNACT2*-*RASGEF1A* region for genetic heterogeneity was plotted, patients were not divided into subgroups with specific genotypes, showing a single cluster (Figure S5). Therefore, it is suggested that there may be no genetic heterogeneity in these *RET*-*CSGALNACT2*-*RASGEF1A* genomic regions showing significant associations with HSCR.

Finally, several well-known HSCR- and/or enteric development-related genes (*EDNRB*, *GDNF*, *NRTN*, *SOX10*, *PHOX2B*, etc.) [1], [2], [26] showed no significant association signals in this study (*_corr_P*>0.05, Table S8). Only, SNPs of *EDNRB* and *ECE1* revealed nominal associations. The lack of associations in *NRTN* and *SOX10* is consistent with reports that have conflicting results [27], [28]. For *EDN3* and *ZFHX1B*, the genetic diversity or the complex phenotypes of patients with combined clinical features might affect no replication of associations in this study [1], [29], [30].

This study found a small amount of inflation of test statistics as the genomic control inflation factor is around 1.07. We applied the linear mixed models (FaST-LMM) to correct for hidden relatedness and found that inflation factor becomes 1.015. This indicates that the linear mixed models successfully removed effects of hidden relatedness as our inflation factor is close to 1. It was also found that *P*-values of most of significant associations detected before correcting for relatedness were consistent with *P*-values after applying the linear mixed models.

In conclusion, our preliminary findings suggest that new potential susceptibility loci, including *SLC6A20*, *RORA*, and *ABCC9* under adjusting the *RET*-*CSGALNACT2*-*RASGEF1A* and *NRG1* regions, may be related to the development of HSCR or ENS-related disorders. However, there are several limitations of this study, such as insufficient sample size and lack of replication. In the case of statistical power of the sample size, it was calculated as 58.5% due to the low numbers of subjects (in particular, L-HSCR and TCA cases due to the rareness of the conditions). Therefore, further replication study with independent samples and functional evaluations of candidate genes are required. In order to share data for further meta-analysis elsewhere, the summary statistics (SNP ID, chromosome, position, allele variation, MAF, OR with 95% CI, and *P*-value) for SNPs with association signals (42,447 SNPs, *_raw_P*<0.05) were summarized in Table S9. In addition, further observation on differential expressions of *RET*, *CSGALNACT2*, and *RASGEF1A* among HSCR subgroups may help our understanding of HSCR pathogenesis and/or ENS development.

## Supporting Information

Figure S1 **The result of principal component analysis.**
(DOC)

Figure S2 **Q-Q plot after excluding SNPs in the** ***RET*** **-** ***CSGALNACT2*** **-** ***RASGEF1A*** **region on chromosome 10q11.2.**
(DOC)

Figure S3 **Regional and LDs of SNPs on** ***RET*** **,** ***CSGALNACT2*** **and** ***RASGEF1A*** **region on chromosome 10.**
(DOC)

Figure S4 **LDs of top three SNPs (kgp4676284, kgp3302846, kgp11922846) and major known risk allele rs2435357 of** ***RET*** **.**
(DOC)

Figure S5 **MDS plot of the** ***RET*** **-** ***CSGALNACT2*** **-** ***RASGEF1A*** **region on chromosome 10 that shows Bonferroni-corrected significances for the HSCR association.**
(DOC)

Table S1 **Top 100 SNPs from association in GWAS analysis.**
(DOC)

Table S2 ***NRG1*** **SNPs with significance (GWAS Raw** ***P*** **<0.05 in this study.**
(DOC)

Table S3 **SNPs with genome-wide significance except for** ***RET-CSGALNACT2-RASGEF1A*** **genomic region.**
(DOC)

Table S4 **List of all SNPs of** ***SLC6A20*** **,** ***RORA*** **, and** ***ABCC9*** **in this GWAS.**
(DOC)

Table S5 **Potential genes showing significant associations (** ***_adj_P*** **<10^−4^) with HSCR under adjusted analysis (only SNPs with** ***P*** **<0.05 shown).**
(DOC)

Table S6 **Top 10 SNPs of** ***RET*** **-** ***CSGALNACT2*** **-** ***RASGEF1A*** **region in each subgroup.**
(DOC)

Table S7 **LD of** ***SLC6A20*** **SNPs with significant associations (** ***_adj_P*** **<0.01) with SNPs of** ***LIMD1*** **and** ***SACM1L*** **.**
(DOC)

Table S8 **SNPs (in this GWAS) of previously known HSCR-related genes.**
(DOC)

Table S9 **Summary statistics for SNPs with association signals (42,447 SNPs,** ***_raw_P*** **<0.05).**
(XLSX)
