# Supplementary material for: A Genome-Wide Association Study Identifies Potential Susceptibility Loci for Hirschsprung Disease
Source: PLoS One. 2014 Oct 13;9(10):e110292. doi: 10.1371/journal.pone.0110292 (PMC4195606; doi:10.1371/journal.pone.0110292)
Supplement: Figure S1 — The result of principal component analysis. (DOC) [file pone.0110292.s001.doc]

**Figure S1**


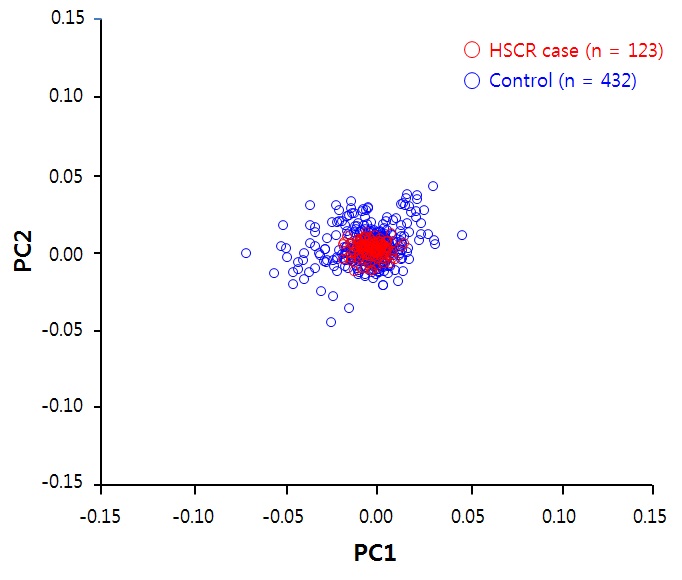


**Figure S1.** The result of principal component analysis. PC1 and PC2 indicate the first and second principal component (PC) of study subjects, respectively.
