# Supplementary material for: A Genome-Wide Association Study Identifies Potential Susceptibility Loci for Hirschsprung Disease
Source: PLoS One. 2014 Oct 13;9(10):e110292. doi: 10.1371/journal.pone.0110292 (PMC4195606; doi:10.1371/journal.pone.0110292)
Supplement: Figure S2 — Q-Q plot after excluding SNPs in the RET - CSGALNACT2 - RASGEF1A region on chromosome 10q11.2. (DOC) [file pone.0110292.s002.doc]

**Figure S2**

**
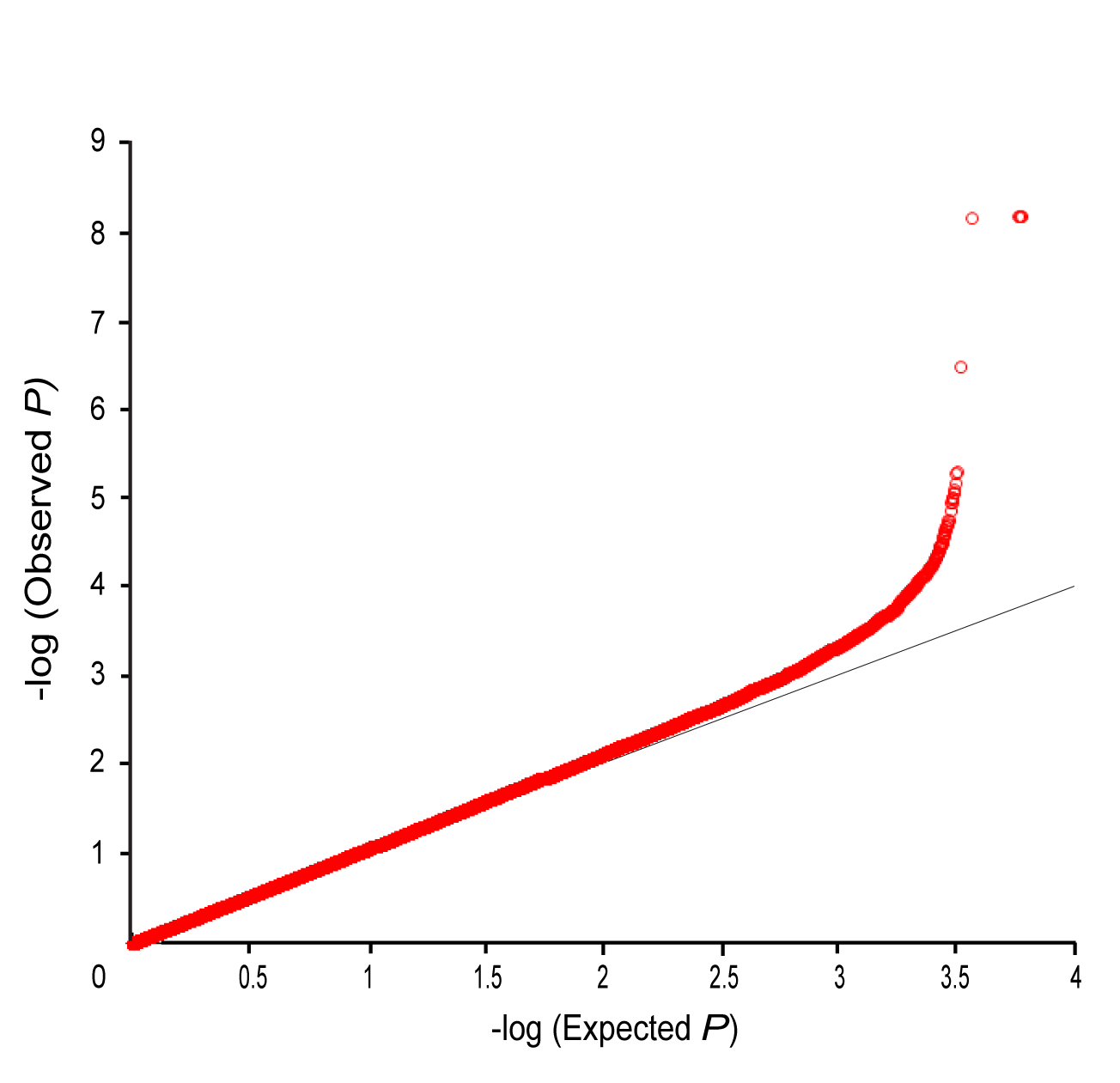
**

**Figure S2.** Q-Q plot after excluding SNPs in the *RET*-*CSGALNACT2*-*RASGEF1A* region on chromosome 10q11.2. The observed *P*-value (y-axis) is compared with the expected *P*-value (x-axis, under null distribution).
