# Supplementary material for: A Genome-Wide Association Study Identifies Potential Susceptibility Loci for Hirschsprung Disease
Source: PLoS One. 2014 Oct 13;9(10):e110292. doi: 10.1371/journal.pone.0110292 (PMC4195606; doi:10.1371/journal.pone.0110292)
Supplement: Figure S3 — Regional and LDs of SNPs on RET , CSGALNACT2 and RASGEF1A region on chromosome 10. (DOC) [file pone.0110292.s003.doc]

**Figure S3**


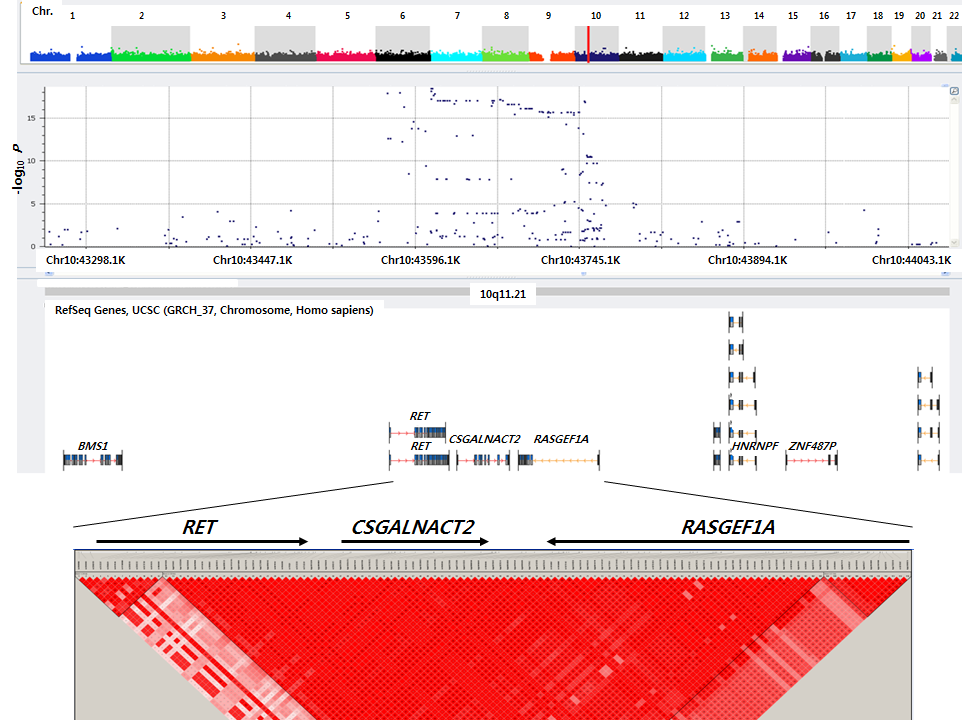


**Figure S3.** Regional and LDs of SNPs on *RET*, *CSGALNACT2* and *RASGEF1A* region on chromosome 10. LDs are indicated with LD coefficient (*r^2^*) between all pairs of biallelic loci.
