# Supplementary material for: A Genome-Wide Association Study Identifies Potential Susceptibility Loci for Hirschsprung Disease
Source: PLoS One. 2014 Oct 13;9(10):e110292. doi: 10.1371/journal.pone.0110292 (PMC4195606; doi:10.1371/journal.pone.0110292)
Supplement: Figure S4 — LDs of top three SNPs (kgp4676284, kgp3302846, kgp11922846) and major known risk allele rs2435357 of RET . (DOC) [file pone.0110292.s004.doc]

**Figure S4**


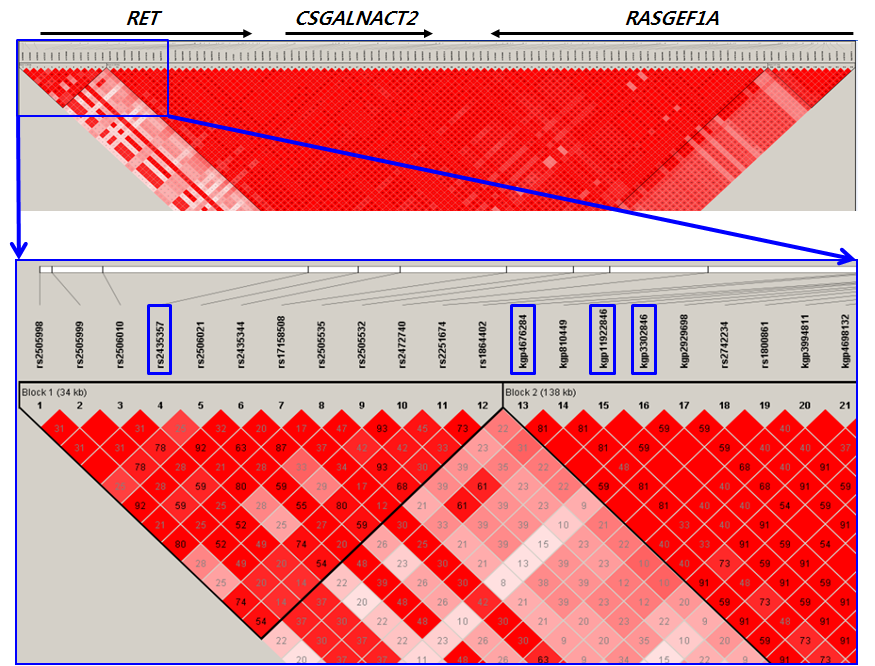


**Figure S4.** LDs of top three SNPs (kgp4676284, kgp3302846, kgp11922846) and major known risk allele rs2435357 of *RET*. Top three intronic SNPs, kgp4676284 (rs1864400), kgp3302846 (rs741968), and kgp11922846 (rs2742233) with higher significances than rs2435357 (major known risk SNP, also known as RET+3, Emison et al. 2005) are in a tight LD but not in LD with rs2435357.
