# Supplementary material for: A Genome-Wide Association Study Identifies Potential Susceptibility Loci for Hirschsprung Disease
Source: PLoS One. 2014 Oct 13;9(10):e110292. doi: 10.1371/journal.pone.0110292 (PMC4195606; doi:10.1371/journal.pone.0110292)
Supplement: Figure S5 — MDS plot of the RET - CSGALNACT2 - RASGEF1A region on chromosome 10 that shows Bonferroni-corrected significances for the HSCR association. (DOC) [file pone.0110292.s005.doc]

**Figure S5**


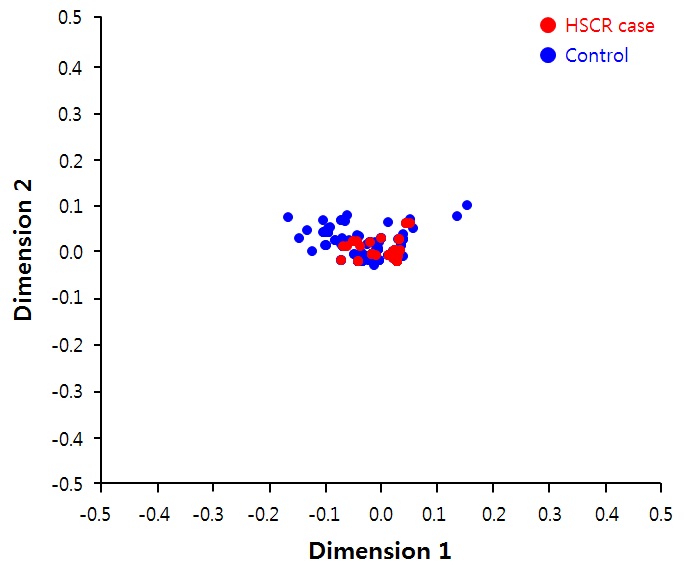


**Figure S5.** **Multidimensional scaling (MDS) plot for genetic heterogeneity of the *RET*-*CSGALNACT2*-*RASGEF1A* region.** MDS plot of the *RET*-*CSGALNACT2*-*RASGEF1A* region on chromosome 10 that shows Bonferroni-corrected significances for the HSCR association.
