# Supplementary material for: A Genome-Wide Association Study Identifies Potential Susceptibility Loci for Hirschsprung Disease
Source: PLoS One. 2014 Oct 13;9(10):e110292. doi: 10.1371/journal.pone.0110292 (PMC4195606; doi:10.1371/journal.pone.0110292)
Supplement: Table S1 — Top 100 SNPs from association in GWAS analysis. (DOC) [file pone.0110292.s006.doc]

**Table S1.** Top 100 SNPs from association in GWAS analysis

| No | SNP ID | Chr. | Position | Gene | Variation | MAF | | *_raw_P*-value* | *_corr_P*-value** |
| --- | --- | --- | --- | --- | --- | --- | --- | --- | --- |
|  |  |  |  |  |  | Case  (n = 123) | Control (n = 432) |  |  |
| 1 | kgp4676284 | 10 | 43610366 | *RET* | G>A | 0.150 | 0.441 | 5.69E-19 | 4.31E-13 |
| 2 | kgp3302846 | 10 | 43611708 | *RET* | A>G | 0.150 | 0.441 | 5.69E-19 | 4.31E-13 |
| 3 | kgp11922846 | 10 | 43610558 | *RET* | C>T | 0.150 | 0.439 | 1.26E-18 | 9.54E-13 |
| 4 | rs2435357 | 10 | 43582056 | *RET* | A>G | 0.199 | 0.499 | 1.86E-18 | 1.41E-12 |
| 5 | rs1800861 | 10 | 43613843 | *RET* | C>A | 0.150 | 0.435 | 2.30E-18 | 1.74E-12 |
| 6 | rs2505998 | 10 | 43570925 | *RET* | A>G | 0.199 | 0.497 | 2.39E-18 | 1.81E-12 |
| 7 | rs2742234 | 10 | 43612609 | *RET* | C>T | 0.152 | 0.436 | 3.81E-18 | 2.89E-12 |
| 8 | kgp6315555 | 10 | 43672776 | *CSGALNACT2* | G>A | 0.159 | 0.440 | 1.17E-17 | 8.86E-12 |
| 9 | kgp9800160 | 10 | 43673731 | *CSGALNACT2* | G>T | 0.159 | 0.440 | 1.17E-17 | 8.86E-12 |
| 10 | kgp810449 | 10 | 43610455 | *RET* | T>C | 0.179 | 0.463 | 1.29E-17 | 9.77E-12 |
| 11 | kgp11512176 | 10 | 43616751 | *RET* | C>A | 0.163 | 0.444 | 1.38E-17 | 1.05E-11 |
| 12 | kgp2737279 | 10 | 43618484 | *RET* | T>C | 0.163 | 0.444 | 1.38E-17 | 1.05E-11 |
| 13 | kgp2737999 | 10 | 43621323 | *RET* | C>G | 0.163 | 0.444 | 1.38E-17 | 1.05E-11 |
| 14 | kgp4996192 | 10 | 43621712 | *RET* | A>G | 0.163 | 0.444 | 1.38E-17 | 1.05E-11 |
| 15 | rs2075912 | 10 | 43622217 | *RET* | T>C | 0.163 | 0.444 | 1.38E-17 | 1.05E-11 |
| 16 | rs2565200 | 10 | 43622933 | *RET* | A>G | 0.163 | 0.444 | 1.38E-17 | 1.05E-11 |
| 17 | rs2742241 | 10 | 43625223 | *RET* | G>A | 0.163 | 0.444 | 1.38E-17 | 1.05E-11 |
| 18 | kgp4557226 | 10 | 43627280 | Intergenic | A>G | 0.163 | 0.444 | 1.38E-17 | 1.05E-11 |
| 19 | kgp7595639 | 10 | 43633248 | Intergenic | A>G | 0.163 | 0.444 | 1.38E-17 | 1.05E-11 |
| 20 | kgp8442950 | 10 | 43642173 | *CSGALNACT2* | G>C | 0.163 | 0.444 | 1.38E-17 | 1.05E-11 |
| 21 | kgp12433252 | 10 | 43645854 | *CSGALNACT2* | C>T | 0.163 | 0.444 | 1.38E-17 | 1.05E-11 |
| 22 | kgp233482 | 10 | 43652445 | *CSGALNACT2* | C>T | 0.163 | 0.444 | 1.38E-17 | 1.05E-11 |
| 23 | kgp11251284 | 10 | 43666016 | *CSGALNACT2* | C>T | 0.163 | 0.444 | 1.38E-17 | 1.05E-11 |
| 24 | kgp6523586 | 10 | 43666767 | *CSGALNACT2* | G>T | 0.163 | 0.444 | 1.38E-17 | 1.05E-11 |
| 25 | kgp8715300 | 10 | 43667398 | *CSGALNACT2* | G>A | 0.163 | 0.444 | 1.38E-17 | 1.05E-11 |
| 26 | kgp3630261 | 10 | 43675671 | *CSGALNACT2* | T>C | 0.163 | 0.443 | 1.54E-17 | 1.17E-11 |
| 27 | kgp8739481 | 10 | 43750144 | *RASGEF1A* | A>G | 0.154 | 0.435 | 1.69E-17 | 1.28E-11 |
| 28 | rs2503853 | 10 | 43750260 | *RASGEF1A* | A>G | 0.154 | 0.434 | 2.46E-17 | 1.86E-11 |
| 29 | kgp4803861 | 10 | 43631698 | Intergenic | C>T | 0.163 | 0.440 | 3.65E-17 | 2.76E-11 |
| 30 | rs1059484 | 10 | 43679800 | *CSGALNACT2* | T>G | 0.167 | 0.444 | 4.22E-17 | 3.20E-11 |
| 31 | kgp9978043 | 10 | 43681251 | Intergenic | G>T | 0.167 | 0.444 | 4.22E-17 | 3.20E-11 |
| 32 | kgp8585205 | 10 | 43683600 | Intergenic | C>T | 0.167 | 0.444 | 4.22E-17 | 3.20E-11 |
| 33 | kgp4319760 | 10 | 43686026 | Intergenic | T>C | 0.167 | 0.444 | 4.22E-17 | 3.20E-11 |
| 34 | rs1254967 | 10 | 43690391 | *RASGEF1A* | A>G | 0.167 | 0.444 | 4.22E-17 | 3.20E-11 |
| 35 | kgp8707302 | 10 | 43665750 | *CSGALNACT2* | A>G | 0.164 | 0.441 | 5.96E-17 | 4.51E-11 |
| 36 | rs2435344 | 10 | 43585874 | *RET* | T>C | 0.211 | 0.497 | 9.30E-17 | 7.04E-11 |
| 37 | rs1254968 | 10 | 43691691 | *RASGEF1A* | T>C | 0.171 | 0.444 | 1.27E-16 | 9.62E-11 |
| 38 | kgp5204826 | 10 | 43696086 | *RASGEF1A* | T>C | 0.171 | 0.444 | 1.27E-16 | 9.62E-11 |
| 39 | kgp3164219 | 10 | 43696992 | *RASGEF1A* | A>G | 0.171 | 0.444 | 1.27E-16 | 9.62E-11 |
| 40 | kgp2195243 | 10 | 43699657 | *RASGEF1A* | T>C | 0.171 | 0.444 | 1.27E-16 | 9.62E-11 |
| 41 | kgp9612770 | 10 | 43700979 | *RASGEF1A* | G>C | 0.171 | 0.444 | 1.27E-16 | 9.62E-11 |
| 42 | kgp11141237 | 10 | 43702143 | *RASGEF1A* | T>C | 0.171 | 0.444 | 1.27E-16 | 9.62E-11 |
| 43 | rs10793424 | 10 | 43717116 | *RASGEF1A* | T>G | 0.171 | 0.440 | 2.84E-16 | 2.15E-10 |
| 44 | kgp12114442 | 10 | 43708998 | *RASGEF1A* | A>G | 0.171 | 0.441 | 2.94E-16 | 2.23E-10 |
| 45 | kgp8341102 | 10 | 43711436 | *RASGEF1A* | T>G | 0.171 | 0.441 | 2.94E-16 | 2.23E-10 |
| 46 | kgp12313493 | 10 | 43712095 | *RASGEF1A* | T>C | 0.171 | 0.440 | 3.29E-16 | 2.49E-10 |
| 47 | kgp8985829 | 10 | 43721462 | *RASGEF1A* | T>C | 0.171 | 0.440 | 3.29E-16 | 2.49E-10 |
| 48 | kgp6617800 | 10 | 43734306 | *RASGEF1A* | G>T | 0.171 | 0.440 | 3.29E-16 | 2.49E-10 |
| 49 | kgp10130865 | 10 | 43734775 | *RASGEF1A* | C>T | 0.171 | 0.440 | 3.29E-16 | 2.49E-10 |
| 50 | kgp2219681 | 10 | 43737767 | *RASGEF1A* | A>C | 0.171 | 0.440 | 3.29E-16 | 2.49E-10 |
| 51 | kgp4223328 | 10 | 43744583 | *RASGEF1A* | A>G | 0.171 | 0.440 | 3.29E-16 | 2.49E-10 |
| 52 | kgp11480513 | 10 | 43744983 | *RASGEF1A* | C>T | 0.171 | 0.440 | 3.29E-16 | 2.49E-10 |
| 53 | rs10793426 | 10 | 43745280 | *RASGEF1A* | A>G | 0.171 | 0.440 | 3.29E-16 | 2.49E-10 |
| 54 | kgp12548077 | 10 | 43718613 | *RASGEF1A* | G>A | 0.171 | 0.439 | 3.67E-16 | 2.78E-10 |
| 55 | kgp8939530 | 10 | 43728780 | *RASGEF1A* | G>A | 0.171 | 0.439 | 3.67E-16 | 2.78E-10 |

**Table S1.** Continued

| 56 | kgp7272806 | 10 | 43740508 | *RASGEF1A* | C>T | 0.171 | 0.439 | 3.67E-16 | 2.78E-10 |
| --- | --- | --- | --- | --- | --- | --- | --- | --- | --- |
| 57 | kgp1567908 | 10 | 43716739 | *RASGEF1A* | A>G | 0.171 | 0.439 | 4.08E-16 | 3.09E-10 |
| 58 | kgp5676435 | 10 | 43686552 | Intergenic | C>T | 0.175 | 0.444 | 6.09E-16 | 4.61E-10 |
| 59 | rs7093409 | 10 | 43740067 | *RASGEF1A* | G>A | 0.171 | 0.436 | 7.53E-16 | 5.70E-10 |
| 60 | kgp11752760 | 10 | 43717423 | *RASGEF1A* | T>C | 0.175 | 0.439 | 1.18E-15 | 8.94E-10 |
| 61 | rs2505532 | 10 | 43594545 | *RET* | G>A | 0.130 | 0.373 | 4.65E-15 | 3.52E-09 |
| 62 | kgp1168258 | 10 | 43733531 | *RASGEF1A* | G>C | 0.187 | 0.447 | 8.27E-15 | 6.26E-09 |
| 63 | kgp12295407 | 10 | 43744860 | *RASGEF1A* | C>G | 0.150 | 0.391 | 2.48E-14 | 1.88E-08 |
| 64 | rs2472740 | 10 | 43598647 | *RET* | C>T | 0.138 | 0.375 | 2.93E-14 | 2.22E-08 |
| 65 | rs2505535 | 10 | 43593043 | *RET* | T>C | 0.236 | 0.500 | 3.03E-14 | 2.29E-08 |
| 66 | rs2251674 | 10 | 43605392 | *RET* | G>A | 0.240 | 0.502 | 5.69E-14 | 4.31E-08 |
| 67 | kgp2984726 | 10 | 43748800 | *RASGEF1A* | G>C | 0.150 | 0.388 | 6.58E-14 | 4.98E-08 |
| 68 | kgp7241473 | 10 | 43648221 | *CSGALNACT2* | G>T | 0.146 | 0.372 | 1.15E-13 | 8.71E-08 |
| 69 | rs2505513 | 10 | 43633537 | *CSGALNACT2* | G>C | 0.146 | 0.370 | 1.43E-13 | 1.08E-07 |
| 70 | rs2505999 | 10 | 43571447 | *RET* | C>T | 0.073 | 0.267 | 2.52E-13 | 1.91E-07 |
| 71 | rs2506010 | 10 | 43573558 | *RET* | C>T | 0.073 | 0.267 | 2.52E-13 | 1.91E-07 |
| 72 | rs2503865 | 10 | 43659536 | *CSGALNACT2* | G>A | 0.150 | 0.370 | 3.74E-13 | 2.83E-07 |
| 73 | rs2506021 | 10 | 43584148 | *RET* | C>T | 0.081 | 0.275 | 5.13E-13 | 3.88E-07 |
| 74 | rs1272142 | 10 | 43695484 | *RASGEF1A* | C>A | 0.154 | 0.370 | 1.07E-12 | 8.10E-07 |
| 75 | kgp171054 | 10 | 43704637 | *RASGEF1A* | G>C | 0.154 | 0.370 | 1.07E-12 | 8.10E-07 |
| 76 | kgp2929698 | 10 | 43611865 | *RET* | T>C | 0.093 | 0.284 | 2.42E-12 | 1.83E-06 |
| 77 | kgp6416882 | 10 | 43617104 | *RET* | A>G | 0.093 | 0.284 | 2.42E-12 | 1.83E-06 |
| 78 | kgp2350154 | 10 | 43620551 | *RET* | G>A | 0.093 | 0.284 | 2.42E-12 | 1.83E-06 |
| 79 | rs2505556 | 10 | 43666623 | *CSGALNACT2* | A>G | 0.093 | 0.284 | 2.42E-12 | 1.83E-06 |
| 80 | kgp3678211 | 10 | 43682162 | Intergenic | T>C | 0.093 | 0.284 | 2.42E-12 | 1.83E-06 |
| 81 | kgp8884660 | 10 | 43682421 | Intergenic | T>C | 0.093 | 0.284 | 2.42E-12 | 1.83E-06 |
| 82 | kgp777042 | 10 | 43685831 | Intergenic | T>C | 0.093 | 0.284 | 2.42E-12 | 1.83E-06 |
| 83 | kgp1938701 | 10 | 43652512 | *CSGALNACT2* | C>G | 0.094 | 0.284 | 3.59E-12 | 2.72E-06 |
| 84 | rs12241923 | 10 | 43713047 | *RASGEF1A* | C>T | 0.154 | 0.362 | 4.94E-12 | 3.74E-06 |
| 85 | rs7915431 | 10 | 43721481 | *RASGEF1A* | G>T | 0.098 | 0.280 | 1.58E-11 | 1.20E-05 |
| 86 | kgp9442037 | 10 | 43718513 | *RASGEF1A* | T>C | 0.098 | 0.279 | 2.10E-11 | 1.59E-05 |
| 87 | rs2460557 | 10 | 43752285 | *RASGEF1A* | G>T | 0.179 | 0.403 | 4.75E-11 | 3.60E-05 |
| 88 | kgp4404044 | 10 | 43752644 | *RASGEF1A* | G>A | 0.179 | 0.403 | 4.75E-11 | 3.60E-05 |
| 89 | kgp696477 | 10 | 43753532 | *RASGEF1A* | T>C | 0.179 | 0.403 | 4.75E-11 | 3.60E-05 |
| 90 | kgp2945005 | 10 | 43754979 | *RASGEF1A* | A>G | 0.179 | 0.403 | 4.75E-11 | 3.60E-05 |
| 91 | kgp3187399 | 10 | 43755095 | *RASGEF1A* | T>G | 0.179 | 0.403 | 4.75E-11 | 3.60E-05 |
| 92 | kgp62679 | 10 | 43755506 | *RASGEF1A* | C>T | 0.179 | 0.403 | 4.75E-11 | 3.60E-05 |
| 93 | kgp7202919 | 10 | 43756440 | *RASGEF1A* | T>C | 0.179 | 0.403 | 4.75E-11 | 3.60E-05 |
| 94 | rs1879307 | 10 | 43757943 | *RASGEF1A* | A>G | 0.187 | 0.403 | 2.50E-10 | 1.89E-04 |
| 95 | kgp2469974 | 10 | 43761347 | *RASGEF1A* | A>G | 0.187 | 0.403 | 2.50E-10 | 1.89E-04 |
| 96 | kgp9027175 | 10 | 43752161 | *RASGEF1A* | T>C | 0.191 | 0.405 | 3.89E-10 | 2.95E-04 |
| 97 | rs2503845 | 10 | 43730473 | *RASGEF1A* | A>C | 0.134 | 0.315 | 5.60E-10 | 4.24E-04 |
| 98 | rs1864402 | 10 | 43605860 | *RET* | A>C | 0.303 | 0.524 | 5.72E-10 | 4.33E-04 |
| 99 | kgp4768713 | 10 | 43729484 | *RASGEF1A* | A>G | 0.134 | 0.314 | 6.15E-10 | 4.66E-04 |
| 100 | kgp4570214 | 10 | 43748422 | *RASGEF1A* | C>T | 0.077 | 0.233 | 1.04E-09 | 7.88E-04 |

**P*-value before the Bonferroni correction; ***P*-value after the Bonferroni correction.

Chr., chromosome; MAF, minor allele frequency; kgp, 1000 Genome Project.
