# Supplementary material for: A Genome-Wide Association Study Identifies Potential Susceptibility Loci for Hirschsprung Disease
Source: PLoS One. 2014 Oct 13;9(10):e110292. doi: 10.1371/journal.pone.0110292 (PMC4195606; doi:10.1371/journal.pone.0110292)
Supplement: Table S2 — NRG1 SNPs with significance (GWAS Raw P <0.05 in this study. (DOC) [file pone.0110292.s007.doc]

**Table S2.** *NRG1* SNPs with significance (GWAS Raw *P* < 0.05 in this study)

| SNP ID | Chr. | Position | Variation | MAF | |  | GWAS | |  | Adjusted analysis** | |
| --- | --- | --- | --- | --- | --- | --- | --- | --- | --- | --- | --- |
|  |  |  |  | Case  (n = 123) | Control  (n = 432) |  | _raw_*P*-value | *_corr_P*-value* |  | OR (95% CI) | *_adj_P*-value |
| rs7822917 | 8 | 32190881 | G>T | 0.195 | 0.137 |  | 0.026 | NS |  | 1.59 (1.02-2.47) | 0.042 |
| rs1685117 | 8 | 32197182 | T>G | 0.439 | 0.374 |  | 0.060 | NS |  | 1.42 (1.01-2.01) | 0.044 |
| rs1685101 | 8 | 32197913 | A>G | 0.435 | 0.356 |  | 0.022 | NS |  | 1.58 (1.11-2.24) | 0.011 |
| rs1487152 | 8 | 32217225 | A>G | 0.272 | 0.211 |  | 0.045 | NS |  | 1.53 (1.04-2.24) | 0.031 |
| rs1626771 | 8 | 32220852 | T>C | 0.463 | 0.383 |  | 0.023 | NS |  | 1.48 (1.06-2.07) | 0.021 |
| rs6992907 | 8 | 32227926 | C>T | 0.272 | 0.206 |  | 0.030 | NS |  | 1.56 (1.06-2.28) | 0.024 |
| rs2347503 | 8 | 32232827 | G>A | 0.459 | 0.382 |  | 0.029 | NS |  | 1.44 (1.03-2.01) | 0.032 |
| rs2347505 | 8 | 32234782 | A>G | 0.463 | 0.383 |  | 0.024 | NS |  | 1.46 (1.05-2.03) | 0.025 |
| rs2347506 | 8 | 32234929 | A>G | 0.362 | 0.295 |  | 0.046 | NS |  | 1.44 (1.02-2.05) | 0.040 |
| rs4352806 | 8 | 32238916 | C>T | 0.439 | 0.354 |  | 0.016 | NS |  | 1.47 (1.06-2.04) | 0.022 |
| rs4035323 | 8 | 32243874 | G>A | 0.435 | 0.354 |  | 0.020 | NS |  | 1.42 (1.02-1.98) | 0.036 |
| rs6986716 | 8 | 32247739 | A>C | 0.272 | 0.216 |  | 0.065 | NS |  | 1.49 (1.01-2.18) | 0.043 |
| rs10100501 | 8 | 32254835 | T>C | 0.443 | 0.353 |  | 0.010 | NS |  | 1.48 (1.06-2.05) | 0.020 |
| rs16879366 | 8 | 32286041 | A>G | 0.118 | 0.059 |  | 0.0034 | NS |  | 2.00 (1.14-3.51) | 0.015 |
| rs11782671 | 8 | 32290601 | C>T | 0.333 | 0.219 |  | 2.99E-04 | NS |  | 1.76 (1.22-2.54) | 0.0023 |
| rs6981660 | 8 | 32298476 | T>C | 0.252 | 0.168 |  | 0.0037 | NS |  | 1.65 (1.11-2.46) | 0.015 |
| rs10954844 | 8 | 32302092 | A>C | 0.130 | 0.073 |  | 0.0067 | NS |  | 1.70 (1.00-2.87) | 0.050 |
| rs6996957 | 8 | 32306984 | T>C | 0.382 | 0.243 |  | 1.97E-05 | NS |  | 1.84 (1.29-2.64) | 7.53E-04 |
| rs17716295 | 8 | 32317917 | C>A | 0.256 | 0.170 |  | 0.0033 | NS |  | 1.67 (1.12-2.49) | 0.012 |
| rs10096770 | 8 | 32325220 | A>G | 0.256 | 0.170 |  | 0.0033 | NS |  | 1.67 (1.12-2.49) | 0.012 |
| rs4733342 | 8 | 32349236 | T>C | 0.134 | 0.071 |  | 0.0023 | NS |  | 1.87 (1.09-3.21) | 0.024 |
| rs11777461 | 8 | 32364970 | C>T | 0.126 | 0.065 |  | 0.0023 | NS |  | 1.98 (1.13-3.49) | 0.017 |
| rs4422736 | 8 | 32370520 | T>C | 0.411 | 0.319 |  | 0.010 | NS |  | 1.40 (1.01-1.96) | 0.044 |
| rs7844425 | 8 | 32375617 | T>G | 0.252 | 0.171 |  | 0.0060 | NS |  | 1.61 (1.08-2.38) | 0.019 |
| rs13282123 | 8 | 32377064 | A>G | 0.126 | 0.068 |  | 0.0043 | NS |  | 1.82 (1.04-3.16) | 0.035 |
| rs6651144 | 8 | 32382668 | T>C | 0.065 | 0.112 |  | 0.028 | NS |  | 0.55 (0.31-0.99) | 0.035 |
| rs12548924 | 8 | 32383339 | T>C | 0.146 | 0.078 |  | 0.0014 | NS |  | 2.02 (1.20-3.43) | 0.0087 |
| rs7000397 | 8 | 32383863 | A>G | 0.254 | 0.166 |  | 0.0028 | NS |  | 1.70 (1.14-2.52) | 0.0090 |
| rs12546350 | 8 | 32397189 | G>A | 0.139 | 0.069 |  | 0.0011 | NS |  | 2.05 (1.19-3.52) | 0.010 |
| rs7826312 | 8 | 32400115 | T>C | 0.260 | 0.168 |  | 0.0018 | NS |  | 1.70 (1.15-2.52) | 0.0082 |
| rs7005606 | 8 | 32401501 | T>G | 0.394 | 0.227 |  | 3.23E-07 | NS |  | 2.21 (1.55-3.17) | 1.02E-05 |
| rs6468119 | 8 | 32401561 | C>T | 0.069 | 0.116 |  | 0.031 | NS |  | 0.54 (0.30-0.96) | 0.029 |
| rs4733130 | 8 | 32406994 | T>C | 0.366 | 0.219 |  | 4.86E-06 | NS |  | 2.20 (1.52-3.18) | 1.98E-05 |
| rs16879552*** | 8 | 32411216 | T>C | 0.451 | 0.341 |  | 0.0019 | NS |  | 1.54 (1.11-2.13) | 0.010 |
| rs7825175 | 8 | 32416274 | G>A | 0.252 | 0.165 |  | 0.0031 | NS |  | 1.73 (1.17-2.56) | 0.0066 |
| rs11776203 | 8 | 32419119 | T>G | 0.114 | 0.053 |  | 0.0016 | NS |  | 2.63 (1.44-4.80) | 0.0015 |
| rs17721043 | 8 | 32436875 | G>A | 0.102 | 0.051 |  | 0.0066 | NS |  | 2.39 (1.29-4.42) | 0.0053 |
| rs16879576 | 8 | 32441235 | A>C | 0.426 | 0.328 |  | 0.0055 | NS |  | 1.52 (1.09-2.11) | 0.012 |
| rs17721282 | 8 | 32443025 | G>A | 0.285 | 0.203 |  | 0.0086 | NS |  | 1.57 (1.08-2.27) | 0.018 |
| rs2466062 | 8 | 32443090 | A>G | 0.285 | 0.203 |  | 0.0086 | NS |  | 1.57 (1.08-2.27) | 0.018 |
| rs12680129 | 8 | 32443145 | G>A | 0.427 | 0.325 |  | 0.0041 | NS |  | 1.55 (1.12-2.14) | 0.0083 |

**P*-value after the Bonferroni correction.

**Analysis after adjustment by sex and 3 SNPs (rs2435357, rs1800860, and rs7078220 on/nearby *RET* in chr. 10) as covariates.

***SNP detected as one of top signals in the previous GWAS of HSCR (*P* = 7.43E-06, Garcia-Barcelo *et al*., Proc Natl Acad Sci U S A, 2009).

Chr., chromosome; MAF, minor allele frequency; OR, odds ratio; CI, confidence interval; NS, not significant.
