# Supplementary material for: A Genome-Wide Association Study Identifies Potential Susceptibility Loci for Hirschsprung Disease
Source: PLoS One. 2014 Oct 13;9(10):e110292. doi: 10.1371/journal.pone.0110292 (PMC4195606; doi:10.1371/journal.pone.0110292)
Supplement: Table S3 — SNPs with genome-wide significance except for RET-CSGALNACT2-RASGEF1A genomic region. (DOC) [file pone.0110292.s008.doc]

***Table S3.*** *SNPs with genome-wide significance except for RET-CSGALNACT2-RASGEF1A genomic region*

| SNP | Chr. | Position | Variation | MAF | | OR (95% CI) | *_corr_P*-value* |
| --- | --- | --- | --- | --- | --- | --- | --- |
|  |  |  |  | Case | Control |  |  |
|  |  |  |  | (n = 123) | (n = 432) |  |  |
| rs12752277 | 1 | 242955437 | C>T | 0.415 | 0.277 | 3.92 (2.36-6.51) | 0.005 |
| rs2809867 | 1 | 242956230 | G>A | 0.415 | 0.277 | 3.92 (2.36-6.51) | 0.005 |
| rs36019094 | 5 | 40237374 | C>A | 0.415 | 0.277 | 3.92 (2.36-6.51) | 0.005 |
| rs35198051 | 11 | 70845204 | G>A | 0.415 | 0.277 | 3.92 (2.36-6.51) | 0.005 |
| rs12739262 | 1 | 202391743 | C>T | 0.415 | 0.277 | 3.92 (2.36-6.50) | 0.005 |

**P*-value after the Bonferroni correction.

Chr., chromosome; MAF, minor allele frequency; OR, odds ratio; CI, confidence interval.
