# Supplementary material for: A Genome-Wide Association Study Identifies Potential Susceptibility Loci for Hirschsprung Disease
Source: PLoS One. 2014 Oct 13;9(10):e110292. doi: 10.1371/journal.pone.0110292 (PMC4195606; doi:10.1371/journal.pone.0110292)
Supplement: Table S4 — List of all SNPs of SLC6A20 , RORA , and ABCC9 in this GWAS. (DOC) [file pone.0110292.s009.doc]

**Table S4.** List of all SNPs of *SLC6A20*, *RORA*, and *ABCC9* in this GWAS

| Gene | SNP ID | Chr.:Position | Variation | MAF | |  | GWAS | |  | Adjusted analysis** | |
| --- | --- | --- | --- | --- | --- | --- | --- | --- | --- | --- | --- |
|  |  |  |  | Case  (n = 123) | Control  (n = 432) |  | _raw_*P*-value | *_corr_P*-value* |  | OR (95% CI) | *_adj_P*-value |
| *SLC6A20* | rs2251109 | 3:45796951 | G>A | 0.167 | 0.188 |  | 0.46 | NS |  | 0.81 (0.53-1.24) | 0.32 |
|  | rs2742388 | 3:45797785 | G>A | 0.098 | 0.090 |  | 0.72 | NS |  | 1.08 (0.60-1.92) | 0.80 |
|  | rs2251347 | 3:45799871 | A>G | 0.098 | 0.082 |  | 0.45 | NS |  | 1.27 (0.70-2.33) | 0.43 |
|  | rs2191028 | 3:45803585 | G>A | 0.142 | 0.172 |  | 0.26 | NS |  | 0.85 (0.54-1.34) | 0.47 |
|  | rs2286489 | 3:45804069 | A>G | 0.321 | 0.360 |  | 0.25 | NS |  | 0.91 (0.64-1.30) | 0.62 |
|  | rs2191027 | 3:45804391 | G>A | 0.004 | 0.041 |  | 6.87E-04 | NS |  | 0.07 (0.01-0.53) | 1.27E-04 |
|  | rs6770261 | 3:45804734 | C>T | 0.069 | 0.142 |  | 9.27E-04 | NS |  | 0.42 (0.23-0.76) | 0.0026 |
|  | rs10461016 | 3:45808701 | T>C | 0.081 | 0.138 |  | 0.014 | NS |  | 0.61 (0.36-1.06) | 0.073 |
|  | rs4299518 | 3:45809273 | T>C | 0.004 | 0.053 |  | 6.34E-05 | NS |  | 0.05 (0.01-0.36) | 2.71E-06 |
|  | rs764177 | 3:45809449 | C>A | 0.309 | 0.396 |  | 0.012 | NS |  | 0.69 (0.49-0.99) | 0.040 |
|  | rs2108917 | 3:45809843 | C>T | 0.171 | 0.226 |  | 0.053 | NS |  | 0.69 (0.45-1.06) | 0.089 |
|  | rs720625 | 3:45810094 | G>A | 0.220 | 0.325 |  | 0.0012 | NS |  | 0.59 (0.40-0.88) | 0.0071 |
|  | rs758387 | 3:45813025 | T>C | 0.220 | 0.320 |  | 0.0022 | NS |  | 0.59 (0.40-0.87) | 0.0065 |
|  | rs2276858 | 3:45813993 | C>T | 0.130 | 0.133 |  | 0.90 | NS |  | 1.12 (0.68-1.83) | 0.66 |
|  | rs13062383 | 3:45816614 | C>T | 0.069 | 0.110 |  | 0.052 | NS |  | 0.69 (0.38-1.25) | 0.21 |
|  | rs758386 | 3:45817418 | C>T | 0.098 | 0.089 |  | 0.69 | NS |  | 1.10 (0.62-1.95) | 0.74 |
|  | rs2531748 | 3:45819837 | C>T | 0.317 | 0.336 |  | 0.59 | NS |  | 0.93 (0.66-1.31) | 0.69 |
|  | rs13067466 | 3:45821244 | T>G | 0.195 | 0.175 |  | 0.47 | NS |  | 1.27 (0.84-1.92) | 0.26 |
|  | rs2742399 | 3:45821573 | G>C | 0.004 | 0.008 |  | 0.48 | NS |  | 0.43 (0.04-4.22) | 0.44 |
|  | rs17078339 | 3:45822437 | A>G | 0.439 | 0.462 |  | 0.51 | NS |  | 0.72 (0.51-1.02) | 0.063 |
|  | rs2531747 | 3:45828586 | G>A | 0.435 | 0.323 |  | 0.0013 | NS |  | 1.96 (1.38-2.79) | 1.30E-04 |
|  | rs9848415 | 3:45828841 | G>A | 0.240 | 0.266 |  | 0.41 | NS |  | 0.71 (0.49-1.05) | 0.078 |
|  | rs2159272 | 3:45829995 | C>T | 0.472 | 0.360 |  | 0.0017 | NS |  | 2.09 (1.47-2.97) | 2.66E-05 |
|  | rs7644870 | 3:45830886 | A>C | 0.423 | 0.464 |  | 0.23 | NS |  | 0.65 (0.45-0.92) | 0.014 |
|  | rs12488144 | 3:45831442 | A>C | 0.203 | 0.166 |  | 0.17 | NS |  | 1.63 (1.06-2.51) | 0.027 |
|  | rs13064991 | 3:45834811 | G>T | 0.382 | 0.444 |  | 0.085 | NS |  | 0.66 (0.47-0.93) | 0.017 |
|  | rs2271615 | 3:45837886 | G>C | 0.122 | 0.069 |  | 0.0080 | NS |  | 1.94 (1.10-3.41) | 0.022 |
| *RORA* | rs3743265 | 15:60821000 | A>G | 0.053 | 0.079 |  | 0.15 | NS |  | 0.46 (0.23-0.94) | 0.025 |
|  | rs10519052 | 15:60823417 | T>C | 0.073 | 0.106 |  | 0.11 | NS |  | 0.53 (0.29-0.98) | 0.037 |
|  | rs2162069 | 15:60861075 | A>G | 0.024 | 0.007 |  | 0.032 | NS |  | 4.32 (1.04-17.91) | 0.044 |
|  | rs3784609 | 15:60910550 | G>A | 0.114 | 0.065 |  | 0.019 | NS |  | 1.84 (1.05-3.21) | 0.035 |
|  | rs1834336 | 15:60935854 | A>G | 0.398 | 0.321 |  | 0.026 | NS |  | 1.45 (1.03-2.05) | 0.033 |
|  | rs17204367 | 15:61040486 | A>G | 0.398 | 0.498 |  | 0.0055 | NS |  | 0.63 (0.45-0.88) | 0.0061 |
|  | rs1351544 | 15:61042867 | G>T | 0.354 | 0.213 |  | 9.49E-06 | NS |  | 2.30 (1.57-3.36) | 1.26E-05 |
|  | rs8025324 | 15:61043378 | G>A | 0.354 | 0.213 |  | 9.49E-06 | NS |  | 2.30 (1.57-3.36) | 1.26E-05 |
|  | rs1318929 | 15:61045030 | T>C | 0.524 | 0.440 |  | 0.018 | NS |  | 1.49 (1.06-2.09) | 0.021 |
|  | rs9920560 | 15:61047930 | C>A | 0.358 | 0.219 |  | 1.74E-05 | NS |  | 2.22 (1.52-3.25) | 2.37E-05 |
|  | rs7183955 | 15:61049569 | A>C | 0.358 | 0.220 |  | 1.88E-05 | NS |  | 2.20 (1.51-3.21) | 3.09E-05 |
|  | rs2279296 | 15:61057064 | T>C | 0.159 | 0.222 |  | 0.025 | NS |  | 0.63 (0.41-0.97) | 0.033 |
|  | rs2279295 | 15:61057225 | T>C | 0.175 | 0.242 |  | 0.022 | NS |  | 0.63 (0.42-0.96) | 0.028 |
|  | rs2279293 | 15:61057357 | G>C | 0.260 | 0.135 |  | 8.38E-06 | NS |  | 2.05 (1.36-3.11) | 6.21E-04 |
|  | rs17204426 | 15:61060230 | T>G | 0.179 | 0.244 |  | 0.027 | NS |  | 0.65 (0.43-0.98) | 0.037 |
|  | rs11071554 | 15:61060956 | T>C | 0.159 | 0.222 |  | 0.027 | NS |  | 0.63 (0.41-0.97) | 0.033 |
|  | rs11637671 | 15:61065607 | A>G | 0.260 | 0.150 |  | 1.23E-04 | NS |  | 1.88 (1.25-2.81) | 0.0023 |
|  | rs10519067 | 15:61068347 | G>A | 0.256 | 0.140 |  | 2.65E-05 | NS |  | 2.14 (1.40-3.26) | 3.88E-04 |
|  | rs11071557 | 15:61068954 | T>C | 0.268 | 0.172 |  | 8.01E-04 | NS |  | 1.81 (1.20-2.73) | 0.0044 |
|  | rs922782 | 15:61070183 | A>C | 0.211 | 0.280 |  | 0.028 | NS |  | 0.65 (0.44-0.97) | 0.029 |
|  | rs2899662 | 15:61076219 | C>T | 0.134 | 0.186 |  | 0.051 | NS |  | 0.55 (0.34-0.88) | 0.0092 |
|  | rs12915776 | 15:61079377 | G>A | 0.256 | 0.206 |  | 0.085 | NS |  | 1.53 (1.01-2.30) | 0.042 |
|  | rs16943131 | 15:61104895 | T>C | 0.049 | 0.089 |  | 0.031 | NS |  | 0.47 (0.24-0.94) | 0.023 |
|  | rs6494226 | 15:61105886 | C>T | 0.049 | 0.088 |  | 0.036 | NS |  | 0.48 (0.24-0.95) | 0.025 |
|  | rs7171681 | 15:61111328 | T>C | 0.154 | 0.203 |  | 0.082 | NS |  | 0.63 (0.40-0.98) | 0.037 |
|  | rs12439995 | 15:61120993 | C>G | 0.397 | 0.324 |  | 0.030 | NS |  | 1.44 (1.00-2.07) | 0.048 |
|  | rs4775301 | 15:61126859 | T>C | 0.276 | 0.346 |  | 0.033 | NS |  | 0.67 (0.46-0.98) | 0.037 |
|  | rs16943299 | 15:61195847 | G>A | 0.118 | 0.166 |  | 0.053 | NS |  | 0.54 (0.32-0.90) | 0.015 |
|  | rs7342684 | 15:61230657 | T>G | 0.179 | 0.252 |  | 0.012 | NS |  | 0.63 (0.42-0.95) | 0.025 |

**Table S4.** Continued

|  | rs12442730 | 15:61237400 | G>A | 0.346 | 0.436 |  | 0.011 | NS |  | 0.70 (0.50-0.98) | 0.034 |
| --- | --- | --- | --- | --- | --- | --- | --- | --- | --- | --- | --- |
|  | rs10438288 | 15:61253741 | A>G | 0.211 | 0.303 |  | 0.0034 | NS |  | 0.54 (0.36-0.80) | 0.0016 |
|  | rs1523527 | 15:61264178 | T>C | 0.187 | 0.265 |  | 0.0074 | NS |  | 0.57 (0.37-0.86) | 0.0066 |
|  | rs1523526 | 15:61264287 | T>G | 0.191 | 0.269 |  | 0.0083 | NS |  | 0.55 (0.37-0.84) | 0.0043 |
|  | rs17237521 | 15:61268282 | C>T | 0.325 | 0.435 |  | 0.0021 | NS |  | 0.64 (0.46-0.89) | 0.0080 |
|  | rs6494237 | 15:61269292 | T>C | 0.167 | 0.226 |  | 0.042 | NS |  | 0.66 (0.43-1.01) | 0.048 |
|  | rs11071577 | 15:61270774 | A>G | 0.230 | 0.284 |  | 0.094 | NS |  | 0.65 (0.44-0.94) | 0.020 |
|  | rs1589702 | 15:61272664 | C>T | 0.329 | 0.413 |  | 0.015 | NS |  | 0.67 (0.47-0.94) | 0.021 |
|  | rs7180208 | 15:61273241 | C>T | 0.378 | 0.456 |  | 0.028 | NS |  | 0.68 (0.49-0.95) | 0.023 |
|  | rs7175830 | 15:61275513 | T>G | 0.488 | 0.379 |  | 0.0026 | NS |  | 1.49 (1.07-2.08) | 0.018 |
|  | rs12101478 | 15:61278222 | T>C | 0.175 | 0.249 |  | 0.013 | NS |  | 0.55 (0.36-0.83) | 0.0032 |
|  | rs7176717 | 15:61284409 | A>G | 0.557 | 0.424 |  | 2.69E-04 | NS |  | 1.57 (1.12-2.18) | 0.0077 |
|  | rs6494242 | 15:61286501 | C>T | 0.163 | 0.245 |  | 0.0054 | NS |  | 0.52 (0.34-0.79) | 0.0014 |
|  | rs7174288 | 15:61287947 | G>A | 0.374 | 0.476 |  | 0.0057 | NS |  | 0.62 (0.45-0.87) | 0.0053 |
|  | rs940224 | 15:61288213 | C>T | 0.488 | 0.381 |  | 0.0027 | NS |  | 1.50 (1.07-2.09) | 0.017 |
|  | rs11637981 | 15:61289622 | G>T | 0.537 | 0.411 |  | 6.13E-04 | NS |  | 1.49 (1.07-2.07) | 0.016 |
|  | rs2030619 | 15:61291680 | T>G | 0.260 | 0.318 |  | 0.071 | NS |  | 0.64 (0.44-0.93) | 0.018 |
|  | rs17237570 | 15:61305612 | C>T | 0.411 | 0.493 |  | 0.019 | NS |  | 0.71 (0.51-0.98) | 0.039 |
|  | rs7168008 | 15:61365803 | C>T | 0.398 | 0.485 |  | 0.016 | NS |  | 0.67 (0.48-0.94) | 0.020 |
|  | rs13329643 | 15:61397660 | C>T | 0.110 | 0.174 |  | 0.012 | NS |  | 0.59 (0.36-0.97) | 0.032 |
|  | rs782916 | 15:61398155 | T>C | 0.020 | 0.055 |  | 0.012 | NS |  | 0.32 (0.12-0.88) | 0.015 |
|  | rs782918 | 15:61399373 | C>T | 0.130 | 0.230 |  | 3.18E-04 | NS |  | 0.50 (0.32-0.78) | 0.0016 |
|  | rs782928 | 15:61407394 | C>T | 0.443 | 0.515 |  | 0.038 | NS |  | 0.71 (0.51-1.00) | 0.048 |
|  | rs782938 | 15:61416631 | C>T | 0.545 | 0.464 |  | 0.020 | NS |  | 1.45 (1.03-2.05) | 0.033 |
|  | rs4365239 | 15:69244680 | C>T | 0.272 | 0.206 |  | 0.036 | NS |  | 1.53 (1.04-2.24) | 0.031 |
|  | rs11072060 | 15:69280089 | G>A | 0.020 | 0.097 |  | 8.55E-06 | NS |  | 0.18 (0.07-0.49) | 4.20E-05 |
|  | rs11072062 | 15:69287571 | G>A | 0.020 | 0.096 |  | 1.08E-05 | NS |  | 0.18 (0.07-0.49) | 4.22E-05 |
|  | rs438866 | 15:69315443 | G>A | 0.020 | 0.093 |  | 2.19E-05 | NS |  | 0.19 (0.07-0.50) | 5.79E-05 |
|  | kgp11303814 | 15:69328226 | C>T | 0.081 | 0.163 |  | 5.25E-04 | NS |  | 0.51 (0.29-0.87) | 0.010 |
| *ABCC9* | rs829060 | 12:21958399 | G>C | 0.163 | 0.158 |  | 0.85 | NS |  | 1.09 (0.68-1.74) | 0.72 |
|  | rs2216525 | 12:21966509 | A>G | 0.008 | 0.013 |  | 0.53 | NS |  | 1.12 (0.20-6.31) | 0.90 |
|  | rs4148679 | 12:21966701 | A>G | 0.183 | 0.163 |  | 0.46 | NS |  | 1.30 (0.83-2.06) | 0.26 |
|  | rs9668454 | 12:21969436 | T>C | 0.293 | 0.229 |  | 0.037 | NS |  | 1.70 (1.14-2.53) | 0.0089 |
|  | rs2638441 | 12:21970019 | G>A | 0.171 | 0.160 |  | 0.68 | NS |  | 1.14 (0.72-1.81) | 0.57 |
|  | rs16924332 | 12:21974228 | T>C | 0.443 | 0.480 |  | 0.28 | NS |  | 0.75 (0.53-1.07) | 0.11 |
|  | rs4148677 | 12:21976428 | G>A | 0.451 | 0.490 |  | 0.26 | NS |  | 0.73 (0.51-1.04) | 0.079 |
|  | rs829074 | 12:21976935 | T>C | 0.191 | 0.175 |  | 0.55 | NS |  | 1.05 (0.68-1.63) | 0.82 |
|  | rs1873638 | 12:21978933 | T>C | 0.195 | 0.181 |  | 0.60 | NS |  | 1.05 (0.68-1.62) | 0.83 |
|  | rs11046200 | 12:21980536 | G>T | 0.134 | 0.120 |  | 0.55 | NS |  | 1.13 (0.68-1.88) | 0.63 |
|  | rs1283809 | 12:21980618 | T>C | 0.061 | 0.060 |  | 0.96 | NS |  | 0.89 (0.44-1.81) | 0.75 |
|  | rs1283810 | 12:21980820 | T>G | 0.211 | 0.186 |  | 0.38 | NS |  | 1.17 (0.76-1.79) | 0.47 |
|  | rs1283811 | 12:21981225 | A>G | 0.211 | 0.186 |  | 0.38 | NS |  | 1.17 (0.76-1.79) | 0.47 |
|  | rs12370561 | 12:21982443 | A>G | 0.122 | 0.115 |  | 0.77 | NS |  | 1.12 (0.66-1.88) | 0.68 |
|  | rs2112080 | 12:21982920 | C>T | 0.337 | 0.251 |  | 0.0082 | NS |  | 1.93 (1.34-2.79) | 3.84E-04 |
|  | rs1283816 | 12:21984711 | T>C | 0.467 | 0.389 |  | 0.026 | NS |  | 1.70 (1.20-2.39) | 0.0022 |
|  | rs1283817 | 12:21984970 | C>A | 0.467 | 0.389 |  | 0.026 | NS |  | 1.70 (1.20-2.39) | 0.0022 |
|  | rs3782667 | 12:21985006 | C>T | 0.350 | 0.271 |  | 0.021 | NS |  | 1.75 (1.23-2.49) | 0.0017 |
|  | rs829068 | 12:21988669 | G>C | 0.471 | 0.390 |  | 0.022 | NS |  | 1.74 (1.23-2.45) | 0.0015 |
|  | rs864360 | 12:21989321 | C>G | 0.382 | 0.295 |  | 0.013 | NS |  | 1.79 (1.27-2.51) | 7.07E-04 |
|  | rs704175 | 12:21991620 | G>A | 0.370 | 0.279 |  | 0.0077 | NS |  | 1.82 (1.29-2.57) | 6.39E-04 |
|  | rs11046205 | 12:21992326 | G>A | 0.118 | 0.126 |  | 0.72 | NS |  | 0.90 (0.54-1.52) | 0.70 |
|  | rs704177 | 12:21993272 | A>T | 0.475 | 0.384 |  | 0.010 | NS |  | 1.78 (1.26-2.52) | 8.10E-04 |
|  | rs704179 | 12:21993620 | G>A | 0.211 | 0.253 |  | 0.16 | NS |  | 0.74 (0.50-1.10) | 0.14 |
|  | rs11046207 | 12:21996045 | T>C | 0.118 | 0.118 |  | 0.99 | NS |  | 1.02 (0.60-1.72) | 0.95 |
|  | rs2307025 | 12:21997942 | A>G | 0.057 | 0.036 |  | 0.17 | NS |  | 1.97 (0.90-4.32) | 0.10 |
|  | rs829079 | 12:21999108 | C>A | 0.447 | 0.361 |  | 0.012 | NS |  | 1.83 (1.28-2.61) | 7.19E-04 |
|  | rs829080 | 12:21999863 | T>C | 0.476 | 0.391 |  | 0.016 | NS |  | 1.81 (1.28-2.57) | 6.52E-04 |
|  | rs2291550 | 12:22001088 | C>T | 0.020 | 0.019 |  | 0.85 | NS |  | 0.92 (0.28-3.03) | 0.89 |
|  | rs1283822 | 12:22001883 | T>G | 0.463 | 0.372 |  | 0.0086 | NS |  | 1.84 (1.29-2.62) | 5.58E-04 |
|  | rs4148674 | 12:22003544 | C>G | 0.357 | 0.266 |  | 0.0072 | NS |  | 1.84 (1.29-2.63) | 6.10E-04 |

**Table S4.** Continued

|  | rs4148673 | 12:22003661 | C>T | 0.333 | 0.244 |  | 0.0062 | NS |  | 1.90 (1.31-2.75) | 5.58E-04 |
| --- | --- | --- | --- | --- | --- | --- | --- | --- | --- | --- | --- |
|  | rs7966768 | 12:22004281 | C>T | 0.053 | 0.035 |  | 0.21 | NS |  | 1.65 (0.75-3.64) | 0.23 |
|  | rs2307024 | 12:22005003 | A>C | 0.354 | 0.255 |  | 0.0026 | NS |  | 1.98 (1.37-2.86) | 1.99E-04 |
|  | rs704187 | 12:22011673 | A>G | 0.370 | 0.266 |  | 0.0019 | NS |  | 1.96 (1.38-2.79) | 1.42E-04 |
|  | rs704189 | 12:22012347 | T>C | 0.373 | 0.266 |  | 0.0016 | NS |  | 1.99 (1.40-2.83) | 1.08E-04 |
|  | rs4148671 | 12:22012422 | G>A | 0.069 | 0.067 |  | 0.92 | NS |  | 1.12 (0.59-2.13) | 0.74 |
|  | rs12230539 | 12:22013213 | A>G | 0.065 | 0.045 |  | 0.22 | NS |  | 1.55 (0.76-3.17) | 0.24 |
|  | rs704190 | 12:22014473 | T>C | 0.362 | 0.249 |  | 6.54E-04 | NS |  | 2.12 (1.47-3.05) | 3.74E-05 |
|  | rs704191 | 12:22015022 | G>A | 0.390 | 0.253 |  | 4.84E-05 | NS |  | 2.12 (1.49-3.03) | 2.16E-05 |
|  | rs704192 | 12:22015114 | C>T | 0.390 | 0.251 |  | 3.38E-05 | NS |  | 2.14 (1.50-3.06) | 1.86E-05 |
|  | rs704193 | 12:22015768 | C>T | 0.370 | 0.452 |  | 0.022 | NS |  | 0.67 (0.48-0.95) | 0.021 |
|  | rs704194 | 12:22016403 | A>T | 0.378 | 0.452 |  | 0.038 | NS |  | 0.69 (0.49-0.96) | 0.029 |
|  | rs697252 | 12:22018164 | A>G | 0.390 | 0.453 |  | 0.082 | NS |  | 0.73 (0.52-1.03) | 0.070 |
|  | rs1283798 | 12:22021195 | C>T | 0.415 | 0.521 |  | 0.0036 | NS |  | 1.58 (1.12-2.23) | 0.0084 |
|  | rs4148669 | 12:22024093 | A>C | 0.386 | 0.253 |  | 6.42E-05 | NS |  | 2.13 (1.49-3.05) | 2.60E-05 |
|  | rs704205 | 12:22030165 | T>G | 0.195 | 0.226 |  | 0.30 | NS |  | 0.71 (0.47-1.07) | 0.10 |
|  | rs1492138 | 12:22030178 | T>A | 0.195 | 0.226 |  | 0.30 | NS |  | 0.71 (0.47-1.07) | 0.10 |
|  | rs4148667 | 12:22031887 | C>T | 0.577 | 0.478 |  | 0.0059 | NS |  | 1.56 (1.11-2.19) | 0.0095 |
|  | rs4148666 | 12:22031991 | G>A | 0.362 | 0.255 |  | 0.0013 | NS |  | 1.92 (1.34-2.74) | 3.17E-04 |
|  | rs12304313 | 12:22032292 | A>C | 0.220 | 0.231 |  | 0.69 | NS |  | 0.81 (0.54-1.22) | 0.31 |
|  | rs7301876 | 12:22034620 | C>T | 0.220 | 0.233 |  | 0.66 | NS |  | 0.80 (0.53-1.21) | 0.29 |
|  | rs4148665 | 12:22035247 | C>T | 0.415 | 0.517 |  | 0.0042 | NS |  | 0.62 (0.44-0.88) | 0.0061 |
|  | rs4148664 | 12:22035425 | T>C | 0.220 | 0.231 |  | 0.69 | NS |  | 0.81 (0.54-1.22) | 0.31 |
|  | rs1356368 | 12:22035873 | G>A | 0.577 | 0.478 |  | 0.0057 | NS |  | 1.56 (1.11-2.19) | 0.0091 |
|  | rs1283802 | 12:22035883 | A>C | 0.415 | 0.516 |  | 0.0049 | NS |  | 0.63 (0.45-0.88) | 0.0060 |
|  | rs1388698 | 12:22036979 | A>G | 0.370 | 0.276 |  | 0.0045 | NS |  | 1.81 (1.26-2.59) | 0.0011 |
|  | rs704206 | 12:22038479 | G>A | 0.553 | 0.455 |  | 0.0062 | NS |  | 1.75 (1.24-2.47) | 0.0013 |
|  | rs1352909 | 12:22040041 | C>T | 0.553 | 0.455 |  | 0.0062 | NS |  | 1.75 (1.24-2.47) | 0.0013 |
|  | rs4762717 | 12:22042512 | G>A | 0.374 | 0.278 |  | 0.0038 | NS |  | 1.81 (1.27-2.58) | 0.0010 |
|  | rs2131136 | 12:22042621 | C>A | 0.171 | 0.174 |  | 0.91 | NS |  | 0.97 (0.61-1.53) | 0.89 |
|  | rs1283807 | 12:22042740 | C>T | 0.553 | 0.457 |  | 0.0073 | NS |  | 1.75 (1.23-2.47) | 0.0014 |
|  | rs2032775 | 12:22044210 | G>A | 0.373 | 0.277 |  | 0.0039 | NS |  | 1.80 (1.26-2.57) | 0.0011 |
|  | rs4148661 | 12:22044843 | G>A | 0.378 | 0.278 |  | 0.0027 | NS |  | 1.85 (1.29-2.64) | 6.34E-04 |
|  | rs2292772 | 12:22045771 | A>G | 0.248 | 0.218 |  | 0.32 | NS |  | 1.20 (0.80-1.79) | 0.38 |
|  | rs1914361 | 12:22045853 | T>C | 0.374 | 0.278 |  | 0.0038 | NS |  | 1.81 (1.27-2.58) | 0.0010 |
|  | rs704215 | 12:22046058 | T>C | 0.248 | 0.218 |  | 0.31 | NS |  | 1.20 (0.80-1.79) | 0.38 |
|  | rs704217 | 12:22047174 | A>G | 0.248 | 0.218 |  | 0.31 | NS |  | 1.20 (0.80-1.79) | 0.38 |
|  | rs10743426 | 12:22051089 | T>C | 0.248 | 0.228 |  | 0.50 | NS |  | 1.20 (0.80-1.79) | 0.38 |
|  | rs4148660 | 12:22059617 | G>C | 0.248 | 0.228 |  | 0.50 | NS |  | 1.20 (0.80-1.79) | 0.38 |
|  | rs11046230 | 12:22060023 | G>A | 0.256 | 0.233 |  | 0.43 | NS |  | 1.26 (0.84-1.88) | 0.27 |
|  | rs1421602 | 12:22061731 | C>T | 0.248 | 0.228 |  | 0.50 | NS |  | 1.20 (0.80-1.79) | 0.38 |
|  | rs4148657 | 12:22062348 | A>T | 0.248 | 0.228 |  | 0.50 | NS |  | 1.20 (0.80-1.79) | 0.38 |
|  | rs4148654 | 12:22063971 | C>T | 0.248 | 0.228 |  | 0.50 | NS |  | 1.20 (0.80-1.79) | 0.38 |
|  | rs4148651 | 12:22067217 | G>A | 0.423 | 0.464 |  | 0.24 | NS |  | 0.89 (0.63-1.25) | 0.51 |
|  | rs3759236 | 12:22068849 | T>G | 0.256 | 0.233 |  | 0.43 | NS |  | 1.26 (0.84-1.88) | 0.27 |
|  | rs4148650 | 12:22069114 | A>C | 0.256 | 0.233 |  | 0.43 | NS |  | 1.26 (0.84-1.88) | 0.27 |
|  | rs11046234 | 12:22072257 | A>G | 0.171 | 0.235 |  | 0.022 | NS |  | 0.66 (0.43-1.03) | 0.064 |
|  | rs10770871 | 12:22075960 | G>T | 0.248 | 0.225 |  | 0.43 | NS |  | 1.18 (0.80-1.76) | 0.41 |
|  | rs2900493 | 12:22078245 | C>G | 0.248 | 0.226 |  | 0.45 | NS |  | 1.18 (0.79-1.76) | 0.41 |
|  | rs2277404 | 12:22078556 | C>T | 0.423 | 0.464 |  | 0.24 | NS |  | 0.89 (0.63-1.25) | 0.51 |
|  | rs2176394 | 12:22078571 | A>T | 0.254 | 0.227 |  | 0.36 | NS |  | 1.26 (0.84-1.88) | 0.26 |
|  | rs2277405 | 12:22078838 | G>T | 0.244 | 0.225 |  | 0.52 | NS |  | 1.16 (0.77-1.73) | 0.48 |
|  | rs870135 | 12:22079263 | A>G | 0.244 | 0.225 |  | 0.51 | NS |  | 1.15 (0.77-1.72) | 0.50 |
|  | rs870134 | 12:22079318 | T>C | 0.252 | 0.229 |  | 0.44 | NS |  | 1.21 (0.81-1.81) | 0.35 |
|  | rs1356370 | 12:22079567 | A>G | 0.250 | 0.229 |  | 0.48 | NS |  | 1.19 (0.79-1.79) | 0.40 |

**P*-value after the Bonferroni correction.

**Analysis after adjustment by sex and 4 SNPs (rs2435357, rs1800860, and rs7078220 on/nearby *RET* and rs16879552 on *NRG1*) as covariates.

In the case of *RORA*, only SNPs with *_adj_P*-value < 0.05 are shown.

Chr., chromosome; MAF, minor allele frequency; OR, odds ratio; CI, confidence interval; kgp, 1000 Genome Project; NS, not significant.
