# Supplementary material for: A Genome-Wide Association Study Identifies Potential Susceptibility Loci for Hirschsprung Disease
Source: PLoS One. 2014 Oct 13;9(10):e110292. doi: 10.1371/journal.pone.0110292 (PMC4195606; doi:10.1371/journal.pone.0110292)
Supplement: Table S5 — Potential genes showing significant associations ( adjP <10−4) with HSCR under adjusted analysis (only SNPs with P <0.05 shown). (DOC) [file pone.0110292.s010.doc]

**Table S5.** Potential genes showing significant associations (*_adj_P* < 10^-4^) with HSCR under adjusted analysis (only SNPs with *P* < 0.05 shown)

| Gene | SNP ID | Chr.:Position | Variation | MAF | |  | GWAS | |  | Adjusted analysis** | |
| --- | --- | --- | --- | --- | --- | --- | --- | --- | --- | --- | --- |
|  |  |  |  | Case  (n = 123) | Control  (n = 432) |  | *_raw_P*-value | *_corr_P*-value* |  | OR (95% CI) | *_adj_P*-value |
| *ASTN1* | rs227511 | 1:177093387 | T>G | 0.175 | 0.241 |  | 0.020 | NS |  | 0.47 (0.30-0.73) | 4.90E-04 |
|  | rs10753143 | 1:177097241 | A>G | 0.193 | 0.285 |  | 0.0024 | NS |  | 0.44 (0.29-0.68) | 8.89E-05 |
|  | rs10798499 | 1:177097442 | A>C | 0.187 | 0.274 |  | 0.0034 | NS |  | 0.45 (0.29-0.69) | 1.18E-04 |
|  | rs10798500 | 1:177098042 | T>C | 0.114 | 0.196 |  | 0.0017 | NS |  | 0.40 (0.24-0.66) | 1.27E-04 |
|  | rs10047146 | 1:177103690 | T>C | 0.118 | 0.205 |  | 0.0011 | NS |  | 0.39 (0.24-0.64) | 8.09E-05 |
|  | rs10047083 | 1:177104130 | C>T | 0.114 | 0.196 |  | 0.0017 | NS |  | 0.40 (0.24-0.66) | 1.27E-04 |
|  | rs972484 | 1:177106260 | C>T | 0.118 | 0.206 |  | 9.34E-04 | NS |  | 0.39 (0.24-0.64) | 7.63E-05 |
| *FIGN* | rs12692701 | 2:164469186 | G>T | 0.553 | 0.463 |  | 0.011 | NS |  | 1.51 (1.08-2.12) | 0.016 |
|  | rs2028406 | 2:164472105 | G>A | 0.089 | 0.186 |  | 7.05E-05 | NS |  | 0.35 (0.20-0.61) | 6.20E-05 |
|  | rs10445755 | 2:164473446 | G>A | 0.020 | 0.080 |  | 1.88E-04 | NS |  | 0.21 (0.08-0.56) | 2.67E-04 |
| *FRAS1* | rs7695549 | 4:79120256 | C>A | 0.463 | 0.377 |  | 0.012 | NS |  | 1.52 (1.07-2.15) | 0.019 |
|  | rs2866993 | 4:79136032 | A>G | 0.451 | 0.341 |  | 0.0014 | NS |  | 1.72 (1.21-2.43) | 0.0020 |
|  | rs7686101 | 4:79138857 | T>G | 0.317 | 0.396 |  | 0.022 | NS |  | 0.69 (0.49-0.97) | 0.032 |
|  | rs11941469 | 4:79148953 | T>C | 0.248 | 0.321 |  | 0.021 | NS |  | 0.65 (0.44-0.96) | 0.028 |
|  | rs9994502 | 4:79156694 | A>G | 0.366 | 0.415 |  | 0.15 | NS |  | 0.71 (0.50-1.00) | 0.048 |
|  | rs11725082 | 4:79158128 | T>C | 0.415 | 0.302 |  | 0.0012 | NS |  | 1.79 (1.27-2.52) | 7.52E-04 |
|  | rs17003071 | 4:79158715 | G>C | 0.024 | 0.055 |  | 0.045 | NS |  | 0.42 (0.17-1.06) | 0.045 |
|  | rs10010118 | 4:79176094 | T>C | 0.407 | 0.477 |  | 0.044 | NS |  | 0.65 (0.47-0.92) | 0.014 |
|  | rs12510741 | 4:79177408 | G>A | 0.407 | 0.477 |  | 0.044 | NS |  | 0.65 (0.47-0.92) | 0.014 |
|  | rs12505887 | 4:79229609 | G>A | 0.341 | 0.413 |  | 0.038 | NS |  | 0.65 (0.46-0.91) | 0.012 |
|  | rs1385133 | 4:79231933 | C>T | 0.423 | 0.292 |  | 6.82E-05 | NS |  | 2.16 (1.49-3.15) | 4.03E-05 |
|  | rs345518 | 4:79236212 | A>C | 0.341 | 0.415 |  | 0.031 | NS |  | 0.64 (0.45-0.91) | 0.013 |
|  | rs345513 | 4:79240063 | T>C | 0.362 | 0.445 |  | 0.017 | NS |  | 0.63 (0.44-0.89) | 0.0075 |
|  | rs2008485 | 4:79276588 | A>G | 0.150 | 0.219 |  | 0.014 | NS |  | 0.60 (0.39-0.94) | 0.022 |
|  | rs447200 | 4:79280088 | T>C | 0.215 | 0.294 |  | 0.014 | NS |  | 0.64 (0.44-0.93) | 0.018 |
|  | rs1385131 | 4:79282759 | C>T | 0.224 | 0.303 |  | 0.013 | NS |  | 0.64 (0.44-0.93) | 0.019 |
|  | rs6833805 | 4:79284428 | G>T | 0.146 | 0.220 |  | 0.0081 | NS |  | 0.57 (0.37-0.90) | 0.012 |
|  | rs6835769 | 4:79284694 | C>T | 0.146 | 0.220 |  | 0.0077 | NS |  | 0.57 (0.37-0.89) | 0.011 |
|  | rs6837091 | 4:79285958 | T>C | 0.386 | 0.305 |  | 0.017 | NS |  | 1.55 (1.10-2.20) | 0.013 |
|  | rs7687731 | 4:79295156 | A>G | 0.370 | 0.284 |  | 0.010 | NS |  | 1.63 (1.14-2.32) | 0.0065 |
|  | rs12509281 | 4:79401434 | A>G | 0.264 | 0.188 |  | 0.0086 | NS |  | 2.16 (1.42-3.28) | 2.60E-04 |
| *CDKAL1* | rs2064320 | 6:20570142 | C>T | 0.211 | 0.176 |  | 0.22 | NS |  | 1.55 (1.01-2.36) | 0.043 |
|  | rs6907767 | 6:20589341 | A>G | 0.236 | 0.195 |  | 0.16 | NS |  | 1.62 (1.08-2.45) | 0.021 |
|  | rs6935317 | 6:20589762 | C>T | 0.236 | 0.190 |  | 0.11 | NS |  | 1.71 (1.13-2.60) | 0.011 |
|  | rs7758129 | 6:20609241 | A>G | 0.224 | 0.182 |  | 0.15 | NS |  | 1.58 (1.04-2.40) | 0.032 |
|  | rs9465831 | 6:20611926 | G>A | 0.215 | 0.171 |  | 0.11 | NS |  | 1.65 (1.08-2.52) | 0.022 |
|  | rs1569660 | 6:20622753 | A>G | 0.199 | 0.162 |  | 0.18 | NS |  | 1.57 (1.02-2.41) | 0.042 |
|  | rs9465838 | 6:20625491 | T>G | 0.199 | 0.161 |  | 0.17 | NS |  | 1.64 (1.06-2.52) | 0.027 |
|  | kgp10397085 | 6:20629491 | A>G | 0.199 | 0.161 |  | 0.17 | NS |  | 1.63 (1.06-2.52) | 0.027 |
|  | rs9350269 | 6:20649534 | C>T | 0.004 | 0.024 |  | 0.018 | NS |  | 0.12 (0.01-1.01) | 0.014 |
|  | kgp6402915 | 6:20657345 | C>T | 0.207 | 0.176 |  | 0.28 | NS |  | 1.53 (1.00-2.32) | 0.049 |
|  | rs12191898 | 6:20865629 | A>G | 0.061 | 0.088 |  | 0.15 | NS |  | 0.51 (0.27-0.99) | 0.038 |
|  | rs7773189 | 6:20945956 | T>G | 0.459 | 0.401 |  | 0.094 | NS |  | 1.45 (1.02-2.05) | 0.037 |
|  | rs1004172 | 6:20954826 | C>T | 0.341 | 0.282 |  | 0.075 | NS |  | 1.52 (1.06-2.18) | 0.023 |
|  | rs7775523 | 6:20955332 | T>C | 0.346 | 0.286 |  | 0.074 | NS |  | 1.55 (1.08-2.22) | 0.017 |
|  | rs2030081 | 6:20971026 | A>G | 0.350 | 0.426 |  | 0.031 | NS |  | 0.69 (0.49-0.98) | 0.036 |
|  | rs201346 | 6:20989286 | C>T | 0.451 | 0.388 |  | 0.065 | NS |  | 1.50 (1.05-2.12) | 0.023 |
|  | rs989969 | 6:21037940 | G>T | 0.394 | 0.288 |  | 0.0014 | NS |  | 1.82 (1.27-2.61) | 0.0010 |
|  | rs9348455 | 6:21043852 | C>T | 0.362 | 0.249 |  | 3.69E-04 | NS |  | 2.26 (1.54-3.32) | 1.96E-05 |
|  | rs9460592 | 6:21066710 | G>A | 0.533 | 0.447 |  | 0.016 | NS |  | 1.44 (1.03-2.00) | 0.031 |
|  | rs4367364 | 6:21076620 | G>A | 0.398 | 0.337 |  | 0.078 | NS |  | 1.66 (1.17-2.35) | 0.0039 |
|  | rs9366375 | 6:21079573 | T>C | 0.333 | 0.387 |  | 0.11 | NS |  | 0.67 (0.47-0.95) | 0.023 |
|  | rs9350319 | 6:21100044 | A>G | 0.276 | 0.323 |  | 0.16 | NS |  | 0.66 (0.46-0.96) | 0.026 |
|  | rs9358391 | 6:21107109 | C>T | 0.272 | 0.321 |  | 0.14 | NS |  | 0.67 (0.47-0.97) | 0.031 |
|  | rs6923546 | 6:21110928 | A>G | 0.285 | 0.349 |  | 0.052 | NS |  | 0.64 (0.44-0.92) | 0.014 |
|  | rs9356764 | 6:21115316 | G>A | 0.285 | 0.347 |  | 0.062 | NS |  | 0.64 (0.44-0.92) | 0.014 |
|  | rs10946430 | 6:21124521 | C>T | 0.280 | 0.347 |  | 0.048 | NS |  | 0.62 (0.43-0.89) | 0.0087 |
|  | rs6937439 | 6:21130870 | A>G | 0.435 | 0.387 |  | 0.19 | NS |  | 1.43 (1.03-1.98) | 0.034 |
|  | rs2446489 | 6:21136120 | C>T | 0.280 | 0.340 |  | 0.075 | NS |  | 0.65 (0.45-0.93) | 0.017 |
|  | rs9358393 | 6:21138100 | A>G | 0.280 | 0.343 |  | 0.060 | NS |  | 0.63 (0.44-0.91) | 0.011 |
|  | rs898165 | 6:21208429 | C>T | 0.285 | 0.212 |  | 0.023 | NS |  | 1.72 (1.18-2.51) | 0.0051 |
|  | rs9358400 | 6:21213307 | G>A | 0.248 | 0.189 |  | 0.049 | NS |  | 1.68 (1.13-2.50) | 0.011 |
|  | rs10946438 | 6:21216746 | C>T | 0.260 | 0.307 |  | 0.15 | NS |  | 0.67 (0.46-0.97) | 0.032 |
| *DGKB* | rs7780524 | 7:14209139 | T>C | 0.309 | 0.366 |  | 0.10 | NS |  | 0.68 (0.48-0.96) | 0.025 |
|  | rs10233920 | 7:14332387 | A>C | 0.150 | 0.125 |  | 0.32 | NS |  | 1.69 (1.04-2.73) | 0.034 |
|  | rs7797910 | 7:14342387 | C>T | 0.037 | 0.014 |  | 0.031 | NS |  | 3.21 (1.07-9.58) | 0.038 |
|  | rs6944473 | 7:14359852 | T>C | 0.041 | 0.014 |  | 0.013 | NS |  | 3.86 (1.33-11.21) | 0.013 |

**Table S5.** Continued

|  | rs2099282 | 7:14386532 | C>T | 0.028 | 0.003 |  | 0.0011 | NS |  | 31.58 (5.68-175.61) | 5.47E-05 |
| --- | --- | --- | --- | --- | --- | --- | --- | --- | --- | --- | --- |
|  | rs6971925 | 7:14445917 | C>T | 0.049 | 0.084 |  | 0.053 | NS |  | 0.46 (0.23-0.94) | 0.023 |
|  | rs196752 | 7:14476211 | G>T | 0.061 | 0.093 |  | 0.10 | NS |  | 0.51 (0.27-0.98) | 0.033 |
|  | rs10950533 | 7:14697617 | A>C | 0.270 | 0.226 |  | 0.16 | NS |  | 1.48 (1.01-2.17) | 0.044 |
| *DYNC1I1* | rs7798936 | 7:95413253 | G>A | 0.207 | 0.313 |  | 6.29E-04 | NS |  | 0.44 (0.29-0.66) | 4.74E-05 |
|  | rs1485001 | 7:95421568 | C>A | 0.220 | 0.265 |  | 0.14 | NS |  | 0.67 (0.45-0.99) | 0.041 |
|  | rs319312 | 7:95559291 | G>A | 0.199 | 0.126 |  | 0.0045 | NS |  | 1.97 (1.25-3.11) | 0.0034 |
| *SLC39A14* | rs2280522 | 8:22272226 | A>G | 0.033 | 0.092 |  | 0.0012 | NS |  | 0.25 (0.11-0.56) | 9.68E-05 |
|  | rs6558052 | 8:22273687 | T>C | 0.110 | 0.166 |  | 0.029 | NS |  | 0.57 (0.35-0.92) | 0.019 |
|  | rs17060854 | 8:22277911 | C>T | 0.037 | 0.091 |  | 0.0027 | NS |  | 0.29 (0.13-0.62) | 3.54E-04 |
| *PTPRD* | rs10977035 | 9:8393528 | A>G | 0.041 | 0.069 |  | 0.080 | NS |  | 0.47 (0.21-1.01) | 0.043 |
|  | rs10511494 | 9:8416196 | T>C | 0.041 | 0.069 |  | 0.080 | NS |  | 0.47 (0.21-1.01) | 0.043 |
|  | rs7855100 | 9:8445864 | A>C | 0.211 | 0.275 |  | 0.035 | NS |  | 0.67 (0.44-1.00) | 0.047 |
|  | rs2890795 | 9:8447732 | T>C | 0.159 | 0.236 |  | 0.0068 | NS |  | 0.61 (0.39-0.95) | 0.025 |
|  | rs7048949 | 9:8522617 | A>G | 0.012 | 0.034 |  | 0.056 | NS |  | 0.27 (0.08-0.96) | 0.020 |
|  | rs10977299 | 9:8784855 | T>C | 0.268 | 0.339 |  | 0.037 | NS |  | 0.66 (0.46-0.95) | 0.024 |
|  | rs7862354 | 9:8791925 | T>C | 0.093 | 0.154 |  | 0.012 | NS |  | 0.55 (0.32-0.93) | 0.021 |
|  | rs4562389 | 9:8805462 | C>T | 0.333 | 0.264 |  | 0.038 | NS |  | 1.47 (1.04-2.10) | 0.031 |
|  | rs10815957 | 9:8815247 | T>C | 0.484 | 0.424 |  | 0.094 | NS |  | 1.50 (1.08-2.10) | 0.016 |
|  | rs1865343 | 9:8845598 | C>T | 0.341 | 0.257 |  | 0.0069 | NS |  | 1.77 (1.21-2.60) | 0.0033 |
|  | rs4742544 | 9:8848290 | C>T | 0.089 | 0.045 |  | 0.010 | NS |  | 4.11 (2.04-8.27) | 9.41E-05 |
|  | rs10759006 | 9:8855603 | C>T | 0.301 | 0.231 |  | 0.036 | NS |  | 1.69 (1.17-2.44) | 0.0053 |
|  | rs751372 | 9:8856908 | G>A | 0.215 | 0.150 |  | 0.015 | NS |  | 2.06 (1.31-3.24) | 0.0018 |
|  | rs10815977 | 9:8860355 | A>G | 0.354 | 0.274 |  | 0.015 | NS |  | 1.47 (1.02-2.13) | 0.039 |
|  | rs723145 | 9:8867519 | C>T | 0.122 | 0.080 |  | 0.052 | NS |  | 2.25 (1.31-3.87) | 0.0035 |
|  | rs10977362 | 9:8922467 | G>A | 0.244 | 0.205 |  | 0.19 | NS |  | 1.49 (1.01-2.22) | 0.048 |
|  | rs636024 | 9:9029235 | A>G | 0.350 | 0.424 |  | 0.034 | NS |  | 0.67 (0.48-0.95) | 0.023 |
|  | rs10977455 | 9:9049562 | A>G | 0.089 | 0.135 |  | 0.049 | NS |  | 0.54 (0.30-0.94) | 0.025 |
|  | rs7035296 | 9:9049813 | C>A | 0.061 | 0.103 |  | 0.039 | NS |  | 0.47 (0.24-0.91) | 0.018 |
|  | rs10977456 | 9:9050817 | T>C | 0.089 | 0.135 |  | 0.049 | NS |  | 0.54 (0.30-0.94) | 0.025 |
|  | rs324498 | 9:9059545 | A>G | 0.122 | 0.170 |  | 0.058 | NS |  | 0.52 (0.32-0.86) | 0.0081 |
|  | rs12006155 | 9:9061331 | T>C | 0.069 | 0.111 |  | 0.048 | NS |  | 0.50 (0.27-0.93) | 0.022 |
|  | rs10977464 | 9:9064311 | T>C | 0.130 | 0.174 |  | 0.10 | NS |  | 0.62 (0.38-1.01) | 0.047 |
|  | rs1992671 | 9:9146140 | G>A | 0.085 | 0.158 |  | 0.0023 | NS |  | 0.47 (0.27-0.80) | 0.0033 |
|  | rs10121402 | 9:9146300 | T>C | 0.090 | 0.157 |  | 0.0051 | NS |  | 0.50 (0.29-0.84) | 0.0064 |
|  | rs1470433 | 9:9148168 | C>A | 0.085 | 0.159 |  | 0.0021 | NS |  | 0.46 (0.27-0.78) | 0.0025 |
|  | rs7036801 | 9:9155721 | C>T | 0.398 | 0.288 |  | 0.0010 | NS |  | 1.63 (1.15-2.31) | 0.0062 |
|  | rs7021891 | 9:9155874 | A>G | 0.089 | 0.168 |  | 0.0013 | NS |  | 0.46 (0.27-0.78) | 0.0021 |
|  | rs12003567 | 9:9161679 | C>T | 0.207 | 0.123 |  | 0.0018 | NS |  | 2.01 (1.30-3.10) | 0.0016 |
|  | rs10977618 | 9:9252816 | T>C | 0.053 | 0.101 |  | 0.015 | NS |  | 0.52 (0.27-1.02) | 0.045 |
|  | rs10435830 | 9:9380781 | A>G | 0.142 | 0.193 |  | 0.062 | NS |  | 0.63 (0.40-0.97) | 0.033 |
|  | rs10977690 | 9:9381760 | C>T | 0.142 | 0.192 |  | 0.068 | NS |  | 0.63 (0.41-0.98) | 0.036 |
|  | rs10977709 | 9:9385640 | T>C | 0.142 | 0.191 |  | 0.072 | NS |  | 0.63 (0.41-0.99) | 0.038 |
|  | rs10816156 | 9:9643972 | T>C | 0.301 | 0.391 |  | 0.0094 | NS |  | 0.67 (0.47-0.94) | 0.019 |
|  | rs4742609 | 9:9683033 | A>G | 0.297 | 0.383 |  | 0.014 | NS |  | 0.71 (0.51-1.00) | 0.046 |
|  | rs12551242 | 9:9700448 | T>C | 0.488 | 0.413 |  | 0.041 | NS |  | 1.40 (1.00-1.96) | 0.048 |
|  | rs10511526 | 9:9701768 | C>T | 0.427 | 0.360 |  | 0.053 | NS |  | 1.44 (1.03-2.02) | 0.033 |
|  | rs13289361 | 9:9707824 | T>C | 0.402 | 0.345 |  | 0.090 | NS |  | 1.60 (1.13-2.27) | 0.0085 |
|  | rs4628308 | 9:9708107 | C>A | 0.398 | 0.340 |  | 0.086 | NS |  | 1.59 (1.12-2.26) | 0.010 |
|  | rs1768885 | 9:9740949 | A>C | 0.520 | 0.458 |  | 0.087 | NS |  | 1.45 (1.05-2.01) | 0.025 |
|  | rs1408122 | 9:9756868 | G>A | 0.415 | 0.376 |  | 0.27 | NS |  | 1.44 (1.02-2.03) | 0.040 |
|  | rs1174587 | 9:9767971 | A>G | 0.073 | 0.105 |  | 0.11 | NS |  | 0.48 (0.26-0.88) | 0.013 |
|  | rs10977970 | 9:9774194 | C>T | 0.427 | 0.394 |  | 0.34 | NS |  | 1.43 (1.02-2.02) | 0.039 |
|  | rs4742634 | 9:9890962 | T>C | 0.187 | 0.133 |  | 0.042 | NS |  | 1.61 (1.04-2.51) | 0.034 |
|  | rs10978082 | 9:9913373 | C>A | 0.114 | 0.181 |  | 0.012 | NS |  | 0.60 (0.37-0.97) | 0.032 |
|  | rs4461956 | 9:9915186 | A>C | 0.419 | 0.502 |  | 0.019 | NS |  | 0.66 (0.47-0.93) | 0.015 |
|  | rs10958936 | 9:10178870 | G>A | 0.500 | 0.404 |  | 0.0088 | NS |  | 1.44 (1.04-1.99) | 0.026 |
|  | rs16925583 | 9:10236864 | T>G | 0.228 | 0.175 |  | 0.077 | NS |  | 1.54 (1.04-2.28) | 0.033 |
|  | rs2475348 | 9:10266608 | C>T | 0.016 | 0.003 |  | 0.044 | NS |  | 11.08 (1.68-73.32) | 0.012 |
|  | rs2475357 | 9:10270339 | G>T | 0.467 | 0.410 |  | 0.11 | NS |  | 1.41 (1.01-1.96) | 0.043 |
|  | rs1322155 | 9:10270636 | G>A | 0.488 | 0.436 |  | 0.16 | NS |  | 1.42 (1.02-1.98) | 0.037 |
|  | rs13291455 | 9:10275622 | A>G | 0.171 | 0.263 |  | 0.0020 | NS |  | 0.55 (0.36-0.84) | 0.0043 |
|  | rs7874027 | 9:10276316 | A>C | 0.189 | 0.270 |  | 0.0082 | NS |  | 0.63 (0.41-0.94) | 0.022 |
| *TSPAN15* | rs12776158 | 10:71218094 | T>C | 0.073 | 0.124 |  | 0.018 | NS |  | 0.54 (0.29-0.98) | 0.035 |
|  | rs10823378 | 10:71218566 | C>T | 0.081 | 0.035 |  | 0.0043 | NS |  | 4.30 (2.12-8.71) | 6.75E-05 |
|  | rs748275 | 10:71244395 | C>T | 0.033 | 0.014 |  | 0.083 | NS |  | 3.52 (1.31-9.49) | 0.016 |
| *ACCSL* | rs12224245 | 11:44071270 | C>T | 0.085 | 0.050 |  | 0.047 | NS |  | 1.98 (1.06-3.68) | 0.034 |
|  | rs11037843 | 11:44073200 | C>T | 0.093 | 0.051 |  | 0.021 | NS |  | 2.14 (1.17-3.93) | 0.016 |
|  | rs12284962 | 11:44077227 | G>A | 0.041 | 0.121 |  | 6.55E-05 | NS |  | 0.23 (0.11-0.48) | 1.13E-05 |
| *CCND1* | rs1352075 | 11:69457293 | T>C | 0.114 | 0.216 |  | 1.61E-04 | NS |  | 0.40 (0.25-0.65) | 9.12E-05 |
|  | rs649392 | 11:69464793 | A>G | 0.073 | 0.128 |  | 0.014 | NS |  | 0.53 (0.30-0.94) | 0.022 |
|  | rs678653 | 11:69466737 | G>C | 0.073 | 0.126 |  | 0.017 | NS |  | 0.51 (0.29-0.90) | 0.015 |
| *PEX5* | rs12424618 | 12:7344802 | A>G | 0.297 | 0.402 |  | 0.0026 | NS |  | 0.49 (0.34-0.71) | 6.95E-05 |
|  | rs7954087 | 12:7351076 | A>G | 0.565 | 0.478 |  | 0.015 | NS |  | 0.59 (0.42-0.83) | 0.0018 |
| *ZNF175* | rs7260534 | 19:52074474 | G>T | 0.248 | 0.157 |  | 0.0011 | NS |  | 2.36 (1.53-3.63) | 8.26E-05 |
|  | rs3752132 | 19:52074537 | T>C | 0.378 | 0.301 |  | 0.015 | NS |  | 1.70 (1.17-2.46) | 0.0048 |
|  | rs6509552 | 19:52075622 | G>A | 0.333 | 0.251 |  | 0.0088 | NS |  | 1.84 (1.26-2.68) | 0.0013 |
|  | rs2305371 | 19:52076481 | A>C | 0.333 | 0.253 |  | 0.010 | NS |  | 1.84 (1.26-2.68) | 0.0014 |
|  | rs1543922 | 19:52084836 | C>T | 0.191 | 0.128 |  | 0.013 | NS |  | 2.00 (1.26-3.17) | 0.0032 |
|  | rs11667382 | 19:52088418 | G>A | 0.386 | 0.323 |  | 0.061 | NS |  | 1.56 (1.09-2.23) | 0.013 |
|  | kgp21441930 | 19:52089982 | C>G | 0.191 | 0.130 |  | 0.015 | NS |  | 2.00 (1.26-3.16) | 0.0033 |

**Table S5.** Continued

| *DSCAM* | rs8128228 | 21:41473566 | A>G | 0.276 | 0.214 |  | 0.039 | NS |  | 1.66 (1.12-2.46) | 0.012 |
| --- | --- | --- | --- | --- | --- | --- | --- | --- | --- | --- | --- |
|  | rs1011765 | 21:41525052 | G>A | 0.228 | 0.177 |  | 0.069 | NS |  | 1.60 (1.04-2.45) | 0.031 |
|  | rs8132673 | 21:41525276 | G>T | 0.272 | 0.208 |  | 0.025 | NS |  | 1.76 (1.17-2.65) | 0.0069 |
|  | rs2837545 | 21:41645484 | A>C | 0.154 | 0.218 |  | 0.023 | NS |  | 0.61 (0.39-0.95) | 0.026 |
|  | rs12481821 | 21:41650265 | T>C | 0.154 | 0.216 |  | 0.029 | NS |  | 0.62 (0.39-0.97) | 0.031 |
|  | rs8133535 | 21:41664277 | T>C | 0.152 | 0.214 |  | 0.027 | NS |  | 0.61 (0.39-0.96) | 0.027 |
|  | rs1882760 | 21:41688767 | A>G | 0.285 | 0.353 |  | 0.045 | NS |  | 0.68 (0.48-0.97) | 0.033 |
|  | rs2837621 | 21:41793013 | T>C | 0.049 | 0.022 |  | 0.032 | NS |  | 2.81 (1.13-7.01) | 0.029 |
|  | rs718099 | 21:41816354 | C>A | 0.171 | 0.135 |  | 0.16 | NS |  | 1.66 (1.04-2.66) | 0.035 |
|  | rs12626382 | 21:41888392 | T>A | 0.272 | 0.182 |  | 0.0027 | NS |  | 2.20 (1.47-3.31) | 1.31E-04 |
|  | rs12483037 | 21:41891665 | A>C | 0.354 | 0.250 |  | 0.0021 | NS |  | 1.88 (1.31-2.71) | 6.01E-04 |
|  | rs2205132 | 21:41899191 | G>T | 0.301 | 0.196 |  | 6.46E-04 | NS |  | 2.22 (1.49-3.30) | 8.05E-05 |
|  | rs1888506 | 21:41899757 | C>T | 0.447 | 0.343 |  | 0.0041 | NS |  | 1.67 (1.19-2.34) | 0.0028 |
|  | rs2837679 | 21:41902761 | T>C | 0.447 | 0.345 |  | 0.0048 | NS |  | 1.66 (1.19-2.33) | 0.0029 |
|  | rs1882782 | 21:41902992 | G>A | 0.447 | 0.347 |  | 0.0059 | NS |  | 1.63 (1.17-2.29) | 0.0038 |
|  | rs2837682 | 21:41904492 | T>G | 0.358 | 0.251 |  | 0.0015 | NS |  | 1.89 (1.31-2.72) | 5.63E-04 |
|  | rs8130144 | 21:41910303 | G>A | 0.285 | 0.193 |  | 0.0031 | NS |  | 1.89 (1.28-2.79) | 0.0012 |
|  | rs2837693 | 21:41910362 | G>A | 0.374 | 0.261 |  | 0.0010 | NS |  | 1.74 (1.23-2.47) | 0.0017 |
|  | rs3804024 | 21:41993707 | T>C | 0.093 | 0.135 |  | 0.068 | NS |  | 0.58 (0.34-1.01) | 0.049 |
|  | rs8127260 | 21:42038328 | A>G | 0.004 | 0.014 |  | 0.15 | NS |  | 0.15 (0.02-1.37) | 0.047 |

**P*-value after the Bonferroni correction.

**Analysis after adjustment by sex and 4 SNPs (rs2435357, rs1800860, and rs7078220 on/nearby *RET* in chr. 10 and rs16879552 on *NRG1*) as covariates.

Chr., chromosome; MAF, minor allele frequency; OR, odds ratio; CI, confidence interval; kgp, 1000 Genome Project; NS, not significant.
