# Supplementary material for: A Genome-Wide Association Study Identifies Potential Susceptibility Loci for Hirschsprung Disease
Source: PLoS One. 2014 Oct 13;9(10):e110292. doi: 10.1371/journal.pone.0110292 (PMC4195606; doi:10.1371/journal.pone.0110292)
Supplement: Table S6 — Top 10 SNPs of RET - CSGALNACT2 - RASGEF1A region in each subgroup. (DOC) [file pone.0110292.s011.doc]

**Table S6.** Top 10 SNPs of *RET*-*CSGALNACT2*-*RASGEF1A* region in each subgroup

| Gene | SNP ID | Chr. | Position | Minor allele | MAF | | OR (95% CI) | GWAS | |
| --- | --- | --- | --- | --- | --- | --- | --- | --- | --- |
|  |  |  |  |  | Case  (n = 123) | Control  (n = 432) |  | *_raw_P*-value | *_corr_P*-value* |
| S-HSCR | kgp11512176 | 10 | 43616751 | A | 0.173 | 0.444 | 0.24 (0.15-0.39) | 2.26E-11 | 1.71E-05 |
|  | kgp2737279 | 10 | 43618484 | C | 0.173 | 0.444 | 0.24 (0.15-0.39) | 2.26E-11 | 1.71E-05 |
|  | kgp2737999 | 10 | 43621323 | G | 0.173 | 0.444 | 0.24 (0.15-0.39) | 2.26E-11 | 1.71E-05 |
|  | kgp4996192 | 10 | 43621712 | G | 0.173 | 0.444 | 0.24 (0.15-0.39) | 2.26E-11 | 1.71E-05 |
|  | rs2075912 | 10 | 43622217 | C | 0.173 | 0.444 | 0.24 (0.15-0.39) | 2.26E-11 | 1.71E-05 |
|  | rs2565200 | 10 | 43622933 | G | 0.173 | 0.444 | 0.24 (0.15-0.39) | 2.26E-11 | 1.71E-05 |
|  | rs2742241 | 10 | 43625223 | A | 0.173 | 0.444 | 0.24 (0.15-0.39) | 2.26E-11 | 1.71E-05 |
|  | kgp4557226 | 10 | 43627280 | G | 0.173 | 0.444 | 0.24 (0.15-0.39) | 2.26E-11 | 1.71E-05 |
|  | kgp7595639 | 10 | 43633248 | G | 0.173 | 0.444 | 0.24 (0.15-0.39) | 2.26E-11 | 1.71E-05 |
|  | kgp8442950 | 10 | 43642173 | C | 0.173 | 0.444 | 0.24 (0.15-0.39) | 2.26E-11 | 1.71E-05 |
|  |  |  |  |  |  |  |  |  |  |
| L-HSCR | rs2505532 | 10 | 43594545 | A | 0.048 | 0.373 | 0.07 (0.02-0.24) | 8.80E-10 | 6.66E-04 |
|  | rs2435357 | 10 | 43582056 | G | 0.129 | 0.499 | 0.13 (0.06-0.29) | 8.87E-10 | 6.72E-04 |
|  | rs2505998 | 10 | 43570925 | G | 0.129 | 0.497 | 0.13 (0.06-0.29) | 9.36E-10 | 7.09E-04 |
|  | rs2435344 | 10 | 43585874 | C | 0.129 | 0.497 | 0.13 (0.06-0.29) | 1.09E-09 | 8.25E-04 |
|  | rs2505535 | 10 | 43593043 | C | 0.145 | 0.500 | 0.15 (0.07-0.32) | 5.31E-09 | 4.02E-03 |
|  | rs2472740 | 10 | 43598647 | T | 0.065 | 0.375 | 0.10 (0.04-0.28) | 7.98E-09 | 6.04E-03 |
|  | kgp810449 | 10 | 43610455 | C | 0.129 | 0.463 | 0.15 (0.07-0.33) | 1.34E-08 | 1.01E-02 |
|  | kgp4676284 | 10 | 43610366 | A | 0.113 | 0.441 | 0.15 (0.06-0.33) | 1.95E-08 | 1.48E-02 |
|  | kgp3302846 | 10 | 43611708 | G | 0.113 | 0.441 | 0.15 (0.06-0.33) | 1.95E-08 | 1.48E-02 |
|  | kgp11922846 | 10 | 43610558 | T | 0.113 | 0.439 | 0.15 (0.07-0.34) | 2.64E-08 | 2.00E-02 |
|  |  |  |  |  |  |  |  |  |  |
| TCA | kgp4676284 | 10 | 43610366 | A | 0.118 | 0.441 | 0.15 (0.05-0.44) | 3.03E-05 | NS |
|  | kgp3302846 | 10 | 43611708 | G | 0.118 | 0.441 | 0.15 (0.05-0.44) | 3.03E-05 | NS |
|  | kgp11922846 | 10 | 43610558 | T | 0.118 | 0.439 | 0.15 (0.05-0.45) | 3.62E-05 | NS |
|  | rs2742234 | 10 | 43612609 | T | 0.118 | 0.436 | 0.15 (0.05-0.45) | 4.00E-05 | NS |
|  | rs1800861 | 10 | 43613843 | A | 0.118 | 0.435 | 0.15 (0.05-0.45) | 4.05E-05 | NS |
|  | kgp8739481 | 10 | 43750144 | G | 0.118 | 0.435 | 0.16 (0.06-0.47) | 5.02E-05 | NS |
|  | rs2503853 | 10 | 43750260 | G | 0.118 | 0.434 | 0.16 (0.06-0.47) | 5.45E-05 | NS |
|  | kgp810449 | 10 | 43610455 | C | 0.147 | 0.463 | 0.17 (0.06-0.47) | 5.86E-05 | NS |
|  | kgp1168258 | 10 | 43733531 | C | 0.147 | 0.447 | 0.20 (0.07-0.53) | 0.00017 | NS |
|  | kgp11512176 | 10 | 43616751 | A | 0.147 | 0.444 | 0.20 (0.07-0.53) | 0.00017 | NS |

**P*-value after the Bonferroni correction.

Chr., chromosome; MAF, minor allele frequency; OR, odds ratio; CI, confidence interval; kgp, 1000 Genome Project; NS, not significant.
