# Supplementary material for: A Genome-Wide Association Study Identifies Potential Susceptibility Loci for Hirschsprung Disease
Source: PLoS One. 2014 Oct 13;9(10):e110292. doi: 10.1371/journal.pone.0110292 (PMC4195606; doi:10.1371/journal.pone.0110292)
Supplement: Table S7 — LD of SLC6A20 SNPs with significant associations ( adjP <0.01) with SNPs of LIMD1 and SACM1L . (DOC) [file pone.0110292.s012.doc]

***Table S7.*** *LD of SLC6A20 SNPs with significant associations (_adj_P < 0.01) with SNPs of LIMD1 and* SACM1L

|  |  | *SLC6A20* | | | | | | | | | | | | | | | | | | | |
| --- | --- | --- | --- | --- | --- | --- | --- | --- | --- | --- | --- | --- | --- | --- | --- | --- | --- | --- | --- | --- | --- |
|  |  | rs2191027 | |  | rs6770261 | |  | rs4299518 | |  | rs720625 | |  | rs758387 | |  | rs2531747 | |  | rs2159272 | |
|  |  | lD'l | r^2^ |  | lD'l | r^2^ |  | lD'l | r^2^ |  | lD'l | r^2^ |  | lD'l | r^2^ |  | lD'l | r^2^ |  | lD'l | r^2^ |
| *LIMD1* | rs267237 | 1 | 0.035 |  | 1 | 0.009 |  | 1 | 0.035 |  | 0.175 | 0.013 |  | 0.107 | 0.005 |  | 0.183 | 0.005 |  | 0.291 | 0.011 |
|  | rs267236 | 1 | 0 |  | 1 | 0.008 |  | 1 | 0 |  | 0.140 | 0.008 |  | 0.071 | 0.002 |  | 0.239 | 0.008 |  | 0.285 | 0.010 |
|  | rs2005227 | 1 | 0.005 |  | 0.719 | 0.030 |  | 1 | 0.005 |  | 0.865 | 0.166 |  | 0.861 | 0.165 |  | 0.039 | 0.001 |  | 0.108 | 0.008 |
|  | rs267232 | 1 | 0.001 |  | 0.392 | 0.069 |  | 1 | 0.001 |  | 0.228 | 0.031 |  | 0.228 | 0.031 |  | 0.170 | 0.004 |  | 0.333 | 0.020 |
|  | rs2578693 | 1 | 0.009 |  | 0.739 | 0.081 |  | 1 | 0.009 |  | 0.739 | 0.330 |  | 0.772 | 0.361 |  | 0.179 | 0.011 |  | 0.024 | 0 |
|  | rs2578692 | 1 | 0.001 |  | 0.688 | 0.096 |  | 1 | 0.001 |  | 0.543 | 0.226 |  | 0.543 | 0.226 |  | 0.197 | 0.011 |  | 0.005 | 0 |
|  | rs267210 | 1 | 0.001 |  | 0.684 | 0.093 |  | 1 | 0.001 |  | 0.540 | 0.220 |  | 0.540 | 0.220 |  | 0.183 | 0.009 |  | 0.006 | 0 |
|  | rs1110650 | 1 | 0.015 |  | 0.050 | 0.001 |  | 1 | 0.015 |  | 0.147 | 0.021 |  | 0.132 | 0.017 |  | 0.074 | 0.002 |  | 0.145 | 0.006 |
|  | rs267230 | 1 | 0.003 |  | 1 | 0.100 |  | 1 | 0.003 |  | 0.655 | 0.160 |  | 0.606 | 0.137 |  | 0.078 | 0.003 |  | 0.063 | 0.003 |
|  | rs3774675 | 1 | 0.003 |  | 1 | 0.100 |  | 1 | 0.003 |  | 0.667 | 0.168 |  | 0.619 | 0.145 |  | 0.083 | 0.004 |  | 0.061 | 0.003 |
|  | rs4683131 | 1 | 0.004 |  | 1 | 0.082 |  | 1 | 0.004 |  | 0.906 | 0.256 |  | 0.905 | 0.255 |  | 0.080 | 0.004 |  | 0.064 | 0.004 |
|  | rs893562 | 1 | 0.004 |  | 1 | 0.082 |  | 1 | 0.004 |  | 0.906 | 0.256 |  | 0.905 | 0.255 |  | 0.080 | 0.004 |  | 0.064 | 0.004 |
|  | rs2012762 | 1 | 0 |  | 0.036 | 0.001 |  | 1 | 0 |  | 0.595 | 0.065 |  | 0.731 | 0.098 |  | 0.053 | 0 |  | 0.097 | 0.001 |
|  | rs3796362 | 1 | 0 |  | 1 | 0.008 |  | 1 | 0 |  | 0.817 | 0.269 |  | 0.817 | 0.269 |  | 0.243 | 0.005 |  | 0.395 | 0.016 |
|  | rs7648681 | 1 | 0.001 |  | 0.688 | 0.096 |  | 1 | 0.001 |  | 0.543 | 0.226 |  | 0.543 | 0.226 |  | 0.197 | 0.011 |  | 0.005 | 0 |
|  | rs12493946 | 1 | 0.007 |  | 1 | 0.134 |  | 1 | 0.007 |  | 0.967 | 0.473 |  | 0.967 | 0.473 |  | 0.111 | 0.005 |  | 0.067 | 0.003 |
|  | rs17078175 | 1 | 0 |  | 1 | 0.008 |  | 1 | 0 |  | 0.801 | 0.236 |  | 0.801 | 0.236 |  | 0.094 | 0.001 |  | 0.009 | 0 |
|  | rs1318992 | 1 | 0.001 |  | 0.775 | 0.260 |  | 1 | 0.001 |  | 0.384 | 0.090 |  | 0.384 | 0.090 |  | 0.004 | 0 |  | 0.381 | 0.027 |
|  | rs2742409 | 1 | 0.073 |  | 0.942 | 0.003 |  | 1 | 0.073 |  | 0.522 | 0.055 |  | 0.641 | 0.083 |  | 0.129 | 0.001 |  | 0.078 | 0 |
|  | rs2247771 | 1 | 0.080 |  | 0.903 | 0.003 |  | 1 | 0.080 |  | 0.595 | 0.065 |  | 0.731 | 0.098 |  | 0.180 | 0.001 |  | 0.097 | 0.001 |
|  | rs3796361 | 1 | 0 |  | 1 | 0.008 |  | 1 | 0 |  | 0.818 | 0.270 |  | 0.818 | 0.270 |  | 0.033 | 0 |  | 0.065 | 0 |
|  |  |  |  |  |  |  |  |  |  |  |  |  |  |  |  |  |  |  |  |  |  |
| *SACM1L* | rs2673051 | 1 | 0.016 |  | 0.216 | 0.001 |  | 1 | 0.016 |  | 0.696 | 0.439 |  | 0.696 | 0.439 |  | 0.097 | 0.002 |  | 0.026 | 0 |
|  | rs11707239 | 1 | 0 |  | 0.864 | 0.700 |  | 1 | 0.000 |  | 0.906 | 0.203 |  | 0.906 | 0.203 |  | 0.023 | 0 |  | 0.037 | 0 |
|  | rs2673050 | 1 | 0.016 |  | 0.431 | 0.004 |  | 1 | 0.016 |  | 0.645 | 0.387 |  | 0.645 | 0.387 |  | 0.092 | 0.002 |  | 0.024 | 0 |
|  | rs1877931 | 1 | 0.000 |  | 0.311 | 0.001 |  | 1 | 0 |  | 0.288 | 0.003 |  | 0.288 | 0.003 |  | 0.163 | 0.002 |  | 0.538 | 0.036 |
|  | rs2271619 | 1 | 0 |  | 0.864 | 0.700 |  | 1 | 0 |  | 0.906 | 0.203 |  | 0.906 | 0.203 |  | 0.023 | 0 |  | 0.037 | 0 |
|  | rs2673028 | 1 | 0.020 |  | 1 | 0.015 |  | 1 | 0.020 |  | 0.694 | 0.353 |  | 0.730 | 0.390 |  | 0.061 | 0.001 |  | 0.047 | 0 |
|  | rs13086080 | 1 | 0.001 |  | 1 | 0.015 |  | 1 | 0.001 |  | 0.685 | 0.334 |  | 0.722 | 0.371 |  | 0.019 | 0 |  | 0.049 | 0 |
|  | rs2673062 | 1 | 0.011 |  | 1 | 0.203 |  | 1 | 0.011 |  | 1 | 0.768 |  | 1 | 0.768 |  | 0.073 | 0.001 |  | 0.028 | 0 |
|  | rs7638902 | 1 | 0.007 |  | 1 | 0.127 |  | 1 | 0.007 |  | 1 | 0.480 |  | 1 | 0.480 |  | 0.081 | 0.003 |  | 0.092 | 0.005 |
|  | rs1468542 | 1 | 0.011 |  | 1 | 0.203 |  | 1 | 0.011 |  | 1 | 0.768 |  | 1 | 0.768 |  | 0.073 | 0.001 |  | 0.028 | 0 |
|  | rs1962800 | 1 | 0.012 |  | 1 | 0.211 |  | 1 | 0.012 |  | 1 | 0.781 |  | 1 | 0.781 |  | 0.085 | 0.002 |  | 0.032 | 0 |
|  | rs1019129 | 1 | 0.011 |  | 1 | 0.203 |  | 1 | 0.011 |  | 1 | 0.768 |  | 1 | 0.768 |  | 0.073 | 0.001 |  | 0.028 | 0 |

Linkage disequilibrium (LD) coefficients (l*D'*l and *r^2^*) of *SLC6A20* SNPs with SNPs of *LIMD1* and *SACM1L* are calculated using the Haploview v4.2 software.
