# Supplementary material for: A Genome-Wide Association Study Identifies Potential Susceptibility Loci for Hirschsprung Disease
Source: PLoS One. 2014 Oct 13;9(10):e110292. doi: 10.1371/journal.pone.0110292 (PMC4195606; doi:10.1371/journal.pone.0110292)
Supplement: Table S8 — SNPs (in this GWAS) of previously known HSCR-related genes. (DOC) [file pone.0110292.s013.doc]

**Table S8.** SNPs (in this GWAS) of previously known HSCR-related genes

| Gene | SNP ID | Chr. | Position | Variation | MAF | |  | *P*-values | | |
| --- | --- | --- | --- | --- | --- | --- | --- | --- | --- | --- |
|  |  |  |  |  | Case  (n = 123) | Control  (n = 432) |  | *_raw_P*-value | *_corr_P*-value* | *_adj_P*-value** |
| *GDNF* | rs2973051 | 5 | 37813540 | A>G | 0.415 | 0.450 |  | 0.32 | NS | 0.15 |
|  | rs17379771 | 5 | 37813591 | C>A | 0.309 | 0.319 |  | 0.78 | NS | 0.82 |
|  | rs11111 | 5 | 37814102 | A>G | 0.244 | 0.192 |  | 0.076 | NS | 0.22 |
|  | rs3749692 | 5 | 37814148 | G>A | 0.366 | 0.385 |  | 0.58 | NS | 0.57 |
|  | rs2973049 | 5 | 37818139 | G>A | 0.426 | 0.442 |  | 0.66 | NS | 0.41 |
|  | rs1549250 | 5 | 37821221 | G>T | 0.325 | 0.363 |  | 0.26 | NS | 0.77 |
|  | rs884344 | 5 | 37824240 | A>C | 0.252 | 0.195 |  | 0.051 | NS | 0.17 |
|  | rs2973042 | 5 | 37827759 | A>C | 0.333 | 0.289 |  | 0.18 | NS | 0.53 |
|  | rs2216711 | 5 | 37828844 | T>C | 0.307 | 0.317 |  | 0.77 | NS | 0.66 |
|  | rs12518844 | 5 | 37832759 | G>A | 0.354 | 0.407 |  | 0.13 | NS | 0.63 |
|  | rs3096140 | 5 | 37832833 | T>C | 0.346 | 0.353 |  | 0.83 | NS | 0.71 |
|  | rs10941370 | 5 | 37833419 | T>C | 0.386 | 0.440 |  | 0.13 | NS | 0.60 |
|  | rs2973033 | 5 | 37839633 | A>G | 0.301 | 0.244 |  | 0.075 | NS | 0.45 |
|  | rs2075681 | 5 | 37840243 | C>T | 0.268 | 0.203 |  | 0.030 | NS | 0.12 |
|  | rs2975100 | 5 | 37841045 | C>T | 0.268 | 0.204 |  | 0.032 | NS | 0.12 |
| *EDNRB* | rs1924922 | 13 | 78461133 | T>C | 0.402 | 0.440 |  | 0.29 | NS | 0.35 |
|  | rs4885491 | 13 | 78470350 | G>A | 0.008 | 0.002 |  | 0.22 | NS | 0.18 |
|  | rs3818416 | 13 | 78474468 | G>T | 0.070 | 0.057 |  | 0.45 | NS | 0.33 |
|  | rs3027111 | 13 | 78481868 | T>C | 0.451 | 0.477 |  | 0.48 | NS | 0.60 |
|  | rs3027139 | 13 | 78490067 | G>A | 0.057 | 0.046 |  | 0.50 | NS | 0.50 |
|  | rs12716722 | 13 | 78492856 | A>G | 0.008 | 0.001 |  | 0.10 | NS | 0.027 |
| *ECE1* | rs3026913 | 1 | 21544601 | G>A | 0.211 | 0.252 |  | 0.18 | NS | 0.28 |
|  | rs3026912 | 1 | 21544735 | G>A | 0.211 | 0.252 |  | 0.18 | NS | 0.28 |
|  | rs2038089 | 1 | 21551457 | A>G | 0.358 | 0.346 |  | 0.72 | NS | 0.66 |
|  | rs2038090 | 1 | 21551550 | A>C | 0.146 | 0.093 |  | 0.014 | NS | 0.030 |
|  | rs12562197 | 1 | 21571601 | G>A | 0.183 | 0.123 |  | 0.017 | NS | 0.053 |
|  | rs212515 | 1 | 21575045 | A>C | 0.415 | 0.331 |  | 0.020 | NS | 0.032 |
|  | rs212516 | 1 | 21575456 | C>T | 0.211 | 0.183 |  | 0.32 | NS | 0.13 |
|  | rs3026886 | 1 | 21581021 | G>A | 0.203 | 0.148 |  | 0.037 | NS | 0.19 |
|  | rs2282715 | 1 | 21581765 | G>A | 0.366 | 0.437 |  | 0.038 | NS | 0.091 |
|  | rs3026883 | 1 | 21581979 | G>T | 0.203 | 0.148 |  | 0.037 | NS | 0.19 |
|  | rs212522 | 1 | 21582425 | G>A | 0.211 | 0.179 |  | 0.26 | NS | 0.10 |
|  | rs212524 | 1 | 21583311 | G>A | 0.215 | 0.231 |  | 0.61 | NS | 0.41 |
|  | rs212526 | 1 | 21584941 | G>A | 0.102 | 0.123 |  | 0.35 | NS | 0.034 |
|  | rs212527 | 1 | 21585014 | G>A | 0.211 | 0.177 |  | 0.22 | NS | 0.090 |
|  | rs2282714 | 1 | 21587849 | C>T | 0.480 | 0.441 |  | 0.27 | NS | 0.22 |
|  | rs2774028 | 1 | 21590342 | C>T | 0.175 | 0.230 |  | 0.053 | NS | 0.10 |
|  | rs212534 | 1 | 21590754 | C>T | 0.102 | 0.125 |  | 0.30 | NS | 0.031 |
|  | rs212539 | 1 | 21592392 | C>T | 0.073 | 0.089 |  | 0.42 | NS | 0.078 |
|  | rs212540 | 1 | 21593117 | A>G | 0.089 | 0.120 |  | 0.15 | NS | 0.037 |
|  | rs212541 | 1 | 21593773 | G>A | 0.085 | 0.116 |  | 0.15 | NS | 0.052 |
|  | rs212549 | 1 | 21598377 | G>A | 0.431 | 0.434 |  | 0.93 | NS | 0.91 |
|  | rs4654916 | 1 | 21600677 | G>A | 0.012 | 0.010 |  | 0.81 | NS | 0.80 |
|  | rs213051 | 1 | 21621536 | T>G | 0.455 | 0.444 |  | 0.76 | NS | 0.55 |
|  | rs213057 | 1 | 21627370 | C>T | 0.455 | 0.390 |  | 0.075 | NS | 0.10 |
|  | rs10916959 | 1 | 21631663 | G>A | 0.081 | 0.109 |  | 0.20 | NS | 0.053 |
|  | rs213010 | 1 | 21639167 | A>G | 0.435 | 0.391 |  | 0.23 | NS | 0.27 |
|  | rs169884 | 1 | 21640565 | C>T | 0.423 | 0.380 |  | 0.24 | NS | 0.28 |
|  | rs84853 | 1 | 21641522 | G>A | 0.419 | 0.390 |  | 0.43 | NS | 0.42 |
|  | rs213032 | 1 | 21654735 | T>C | 0.435 | 0.400 |  | 0.35 | NS | 0.30 |
|  | rs213037 | 1 | 21655884 | G>A | 0.435 | 0.400 |  | 0.35 | NS | 0.30 |
|  | rs213039 | 1 | 21657162 | G>T | 0.426 | 0.396 |  | 0.40 | NS | 0.39 |
|  | rs3026815 | 1 | 21664475 | G>A | 0.053 | 0.029 |  | 0.079 | NS | 0.056 |
|  | rs2745251 | 1 | 21674005 | T>C | 0.427 | 0.407 |  | 0.58 | NS | 0.39 |
| *EDN3* | rs11570255 | 20 | 57875916 | G>A | 0.033 | 0.036 |  | 0.79 | NS | 0.98 |
|  | rs260741 | 20 | 57876155 | C>T | 0.333 | 0.295 |  | 0.25 | NS | 0.25 |
|  | rs197174 | 20 | 57878080 | T>C | 0.163 | 0.209 |  | 0.10 | NS | 0.051 |
|  | rs197173 | 20 | 57878591 | C>A | 0.285 | 0.274 |  | 0.74 | NS | 0.75 |
|  | rs2268689 | 20 | 57887918 | T>C | 0.150 | 0.194 |  | 0.10 | NS | 0.16 |
|  | rs2284804 | 20 | 57890199 | A>G | 0.077 | 0.120 |  | 0.051 | NS | 0.080 |
|  | rs2284803 | 20 | 57892300 | C>A | 0.146 | 0.190 |  | 0.10 | NS | 0.14 |
| *SOX10* | rs139883 | 22 | 38369027 | T>C | 0.285 | 0.242 |  | 0.18 | NS | 0.19 |
|  | rs139884 | 22 | 38369976 | G>A | 0.285 | 0.227 |  | 0.069 | NS | 0.11 |
|  | rs139885 | 22 | 38371039 | T>C | 0.285 | 0.242 |  | 0.18 | NS | 0.19 |
|  | rs139886 | 22 | 38371328 | T>C | 0.285 | 0.242 |  | 0.18 | NS | 0.19 |
|  | rs11912587 | 22 | 38371933 | A>C | 0.033 | 0.015 |  | 0.092 | NS | 0.24 |

**Table S8.** Continued

| *NRTN* | rs3763045 | 19 | 5822490 | C>A | 0.114 | 0.105 |  | 0.70 | NS | 0.96 |
| --- | --- | --- | --- | --- | --- | --- | --- | --- | --- | --- |
|  | rs3763046 | 19 | 5823903 | A>G | 0.524 | 0.487 |  | 0.29 | NS | 0.16 |
|  | rs8106995 | 19 | 5827620 | T>C | 0.114 | 0.124 |  | 0.67 | NS | 0.49 |
| *ZEB2* | rs16855324 | 2 | 145147758 | C>T | 0.073 | 0.059 |  | 0.42 | NS | 0.54 |
| (*ZFHX1B*) | rs10185359 | 2 | 145155731 | C>T | 0.146 | 0.125 |  | 0.38 | NS | 0.56 |
|  | rs16823675 | 2 | 145173029 | T>C | 0.183 | 0.183 |  | 1.00 | NS | 0.94 |
|  | rs6748886 | 2 | 145177708 | C>T | 0.073 | 0.059 |  | 0.41 | NS | 0.64 |
|  | rs7600752 | 2 | 145178110 | A>G | 0.362 | 0.334 |  | 0.41 | NS | 0.64 |
|  | rs10192562 | 2 | 145184316 | A>G | 0.106 | 0.093 |  | 0.55 | NS | 0.65 |
|  | rs12614546 | 2 | 145186749 | T>C | 0.358 | 0.335 |  | 0.50 | NS | 0.74 |
|  | rs2288353 | 2 | 145187783 | A>G | 0.285 | 0.274 |  | 0.74 | NS | 0.90 |
|  | rs13013418 | 2 | 145202375 | A>C | 0.061 | 0.043 |  | 0.27 | NS | 0.29 |
|  | rs16823732 | 2 | 145204758 | G>A | 0.110 | 0.094 |  | 0.46 | NS | 0.49 |
|  | rs7568133 | 2 | 145204976 | G>A | 0.179 | 0.191 |  | 0.64 | NS | 0.46 |
|  | rs12105918 | 2 | 145208193 | T>C | 0.110 | 0.095 |  | 0.49 | NS | 0.49 |
|  | rs7597006 | 2 | 145209076 | C>T | 0.340 | 0.328 |  | 0.70 | NS | 0.87 |
|  | rs1427298 | 2 | 145214421 | A>G | 0.362 | 0.351 |  | 0.74 | NS | 0.82 |
|  | rs7599224 | 2 | 145214607 | T>G | 0.362 | 0.351 |  | 0.74 | NS | 0.82 |
|  | rs12327962 | 2 | 145218605 | G>A | 0.138 | 0.138 |  | 0.98 | NS | 0.96 |
|  | rs13002663 | 2 | 145220163 | C>T | 0.280 | 0.279 |  | 0.96 | NS | 0.89 |
|  | rs13403907 | 2 | 145222038 | A>G | 0.313 | 0.280 |  | 0.31 | NS | 0.23 |
|  | rs13382811 | 2 | 145223620 | C>T | 0.283 | 0.246 |  | 0.24 | NS | 0.18 |
|  | rs17677601 | 2 | 145223732 | G>T | 0.142 | 0.178 |  | 0.18 | NS | 0.35 |
|  | rs6717029 | 2 | 145225885 | C>T | 0.138 | 0.171 |  | 0.22 | NS | 0.34 |
|  | rs4662223 | 2 | 145235108 | G>A | 0.163 | 0.190 |  | 0.32 | NS | 0.43 |
|  | rs7600781 | 2 | 145236142 | G>A | 0.443 | 0.458 |  | 0.68 | NS | 0.79 |
|  | rs7573925 | 2 | 145241547 | G>T | 0.004 | 0.001 |  | 0.39 | NS | 0.18 |
|  | rs6755392 | 2 | 145242122 | G>A | 0.443 | 0.458 |  | 0.68 | NS | 0.79 |
|  | rs12691693 | 2 | 145255210 | C>T | 0.508 | 0.497 |  | 0.75 | NS | 0.34 |
|  | rs1035822 | 2 | 145255778 | G>A | 0.122 | 0.097 |  | 0.26 | NS | 0.76 |
|  | rs12471396 | 2 | 145260260 | C>T | 0.122 | 0.086 |  | 0.087 | NS | 0.084 |
|  | rs6740731 | 2 | 145270592 | G>A | 0.358 | 0.394 |  | 0.30 | NS | 0.78 |
|  | rs3806475 | 2 | 145279786 | T>C | 0.455 | 0.477 |  | 0.55 | NS | 0.85 |
|  | rs12691694 | 2 | 145282685 | G>A | 0.484 | 0.471 |  | 0.73 | NS | 0.68 |
| *PHOX2B* | rs6826373 | 4 | 41746460 | C>T | 0.154 | 0.243 |  | 0.0024 | NS | 0.095 |
|  | rs4608840 | 4 | 41749938 | C>T | 0.146 | 0.214 |  | 0.017 | NS | 0.18 |

**P*-value after the Bonferroni correction.

***P*-value after adjustment by sex and 4 SNPs (rs2435357, rs1800860, and rs7078220 on/nearby *RET* and rs16879552 on *NRG1*) as covariates.

Chr., chromosome; MAF, minor allele frequency; NS, not significant.
